# Patient-Proxy and Societal Perspectives of Quality-of-Life Utilities in Children With Cleft Lip and Palate Managed With Surgical Repair vs No Repair in Ethiopia

**DOI:** 10.1001/jamanetworkopen.2022.20900

**Published:** 2022-07-14

**Authors:** Karen Y. Chung, Gebremedhin B. Gebretekle, Andrew Howard, Eleanor Pullenayegum, Mekonen Eshete, Christopher R. Forrest, Beate Sander

**Affiliations:** 1 Division of Plastic, Reconstructive, and Aesthetic Surgery, Department of Surgery, University of Toronto, Toronto, Ontario, Canada; 2 College of Health Sciences, Addis Ababa University, Addis Ababa, Ethiopia; 3 Division of Orthopedic Surgery, Department of Surgery, Hospital for Sick Children, University of Toronto, Toronto, Ontario, Canada; 4 Division of Biostatistics, Child Health Evaluative Services, Hospital for Sick Children, University of Toronto, Toronto, Ontario, Canada; 5 Smile Train Research and Innovation Advisory Council Member and College of Health Sciences, Addis Ababa University Surgical Department, Yekatit 12 Hospital Medical College, Addis Ababa, Ethiopia; 6 Toronto Health Economics and Technology Assessment Collaborative, University Health Network, Toronto, Ontario, Canada; 7 Institute of Health Policy, Management and Evaluation, University of Toronto, Toronto, Ontario, Canada; 8 Public Health Ontario, Windsor, Ontario, Canada; 9 ICES (formerly the Institute for Clinical Evaluative Sciences), Sunnybrook Health Sciences Centre, Toronto, Ontario, Canada; 10 Child Health Evaluative Services, Hospital for Sick Children, University of Toronto, Toronto, Ontario, Canada

## Abstract

**Importance:**

In Ethiopia, more than 70% of infants with cleft lip and/or palate (CL/P) lack access to surgery. Infants who are untreated can experience severe malnutrition and extreme social stigma resulting in abandonment. Utilities are standardized measures of health-related quality of life (HRQOL) that inform health care resource allocation. However, CL/P utilities are missing from low- and middle-income countries (LMICs).

**Objective:**

To elicit utilities for untreated and surgically treated children with CL/P with consideration for social determinants of health from patient-proxy and societal participants.

**Design, Setting, and Participants:**

This cross-sectional study used patient proxies and societal participants in Addis Ababa, Ethiopia, from July 1, 2019, to January 30, 2020. Eligible patient proxies were caregivers of children younger than 18 years with nonsyndromic CL/P who were untreated or received surgery. Proxies were necessary as most patients were 0 to 4 years old and cannot reliably self-report. Eligible societal participants were 18 years and older with no family history of CL/P.

**Exposures:**

Surgical treatment and social determinants of health.

**Main Outcomes and Measures:**

Participants measured utilities using the visual analog scale (VAS), time trade-off (TTO), and standard gamble (SG).

**Results:**

In this study, 312 patient proxies and 135 societal participants were recruited. Mean (SD) utilities for untreated CL/P ranged from 0.57 (0.23) to 0.70 (0.22) from patient proxies and from 0.35 (0.21) to 0.8 (0.23) from societal participants, depending on utility instrument and cleft type. Surgical treatment was associated with a better HRQOL from the patient-proxy perspective (VAS, 0.17; 95% CI, 0.09 to 0.26; TTO, 0.15; 95% CI, 0.05 to 0.25) from the societal perspective (VAS, 0.21; 95% CI, 0.16 to 0.26; TTO, 0.17; 95% CI, 0.13 to 0.22; SG, 0.11; 95% CI, 0.06 to 0.15). Social determinants of health that were associated with patient-proxy utilities were income above the national mean (VAS, 0.10; 95% CI, 0.02 to 0.17; TTO, 0.11; 95% CI, 0.02 to 0.20), and religion (Christian vs other: TTO, −0.10; 95% CI, −0.17 to −0.03). From the societal perspective, the association between treatment and utilities was smaller in females compared with males (TTO, −0.05; 95% CI, −0.10 to −0.01).

**Conclusions and Relevance:**

The findings of this study suggest that CL/P disease severity and surgical impact in Ethiopia were undervalued by previous estimates from high-income countries and were associated with social determinants of health. Utility studies from participants from LMICs are feasible and necessary for representing HRQOL in LMICs and addressing health inequalities.

## Key Points

**Question:**

What are the perspectives of patient proxies and community members about quality-of-life utilities for children with cleft lip and/or palate (CL/P) who receive surgery or do not receive surgery, and are they associated with sociodemographic factors?

**Findings:**

This cross-sectional study of 312 patient proxies and 135 societal participants showed that CL/P disease severity and the effect of surgical repair in Ethiopia were undervalued by previous estimates from high-income countries. Surgical treatment, income, religion, and sex had a significant association with utility-based health-related quality of life.

**Meaning:**

These findings suggest that utilities for CL/P from low-resource settings are necessary, and continuing with the status quo risks further marginalization in health care resource allocation.

## Teaser

This cross-sectional study examines patient-proxy and societal perspectives of quality-of-life utilities for untreated and surgically treated children with cleft lip and palate.

## Introduction

Of the 4.8 billion people in the world who lack access to safe surgical care, cleft lip and/or palate (CL/P) is the most common craniofacial condition affecting this population.^1,2^ Ethiopia is the second most populous country in Africa, with a reported CL/P estimate of 1 in 672 live births; yet, more than 70% of patients with CL/P lack access to surgery.^3,4^ Children who do not receive treatment can experience swallowing difficulties, severe malnutrition, speech deficiencies, and extreme social stigma resulting in abandonment.^5,6^ Surgical treatment of CL/P is reported to significantly improve health-related quality of life (HRQOL).^6,7^

Utilities are standardized HRQOL measures that represent the physical, social, and mental well-being of individuals and populations.^8,9,10^ Utilities can be reported from the patient or societal perspectives and both perspectives are important to consider.^11^ The patient perspective provides firsthand experience of the disease and treatment, while the societal perspective must also be considered as resource allocation decisions affect the health of the entire community.^11^ The primary benefit of utilities is that they allow for a standardized means of comparing the burden of different diseases and the impact of different interventions.^9^ When utilities are incorporated into cost-effectiveness analyses (CEAs), utilities enable policymakers to maximize individual and population health within existing resource constraints.^9,11^

Unfortunately, surgical utilities from low- and middle-income countries (LMICs) are scarce. LMICs, such as Ethiopia, have recently developed national health technology assessment agencies that use utilities to inform policy decisions, and utilities for pharmaceutical interventions in these settings have increased.^12,13,14,15,16,17^ Because of the paucity of utilities for surgical diseases, LMIC surgical CEAs have relied on disability weights. These alternative measures of HRQOL are reported as being universal but are largely derived from high-income settings, lack consideration for local social determinants of health, and omit the patient perspective.^13,18,19,20^

There is a need for CL/P utilities from LMICs to better inform CEAs for more equitable health care resource allocation. The purpose of this study was to elicit utilities for children with CL/P and assess the impact of primary surgical repair and social determinants of health on utility-based HRQOL from the patient and societal perspectives in Ethiopia.

## Methods

This cross-sectional study was approved by the research ethics boards at Addis Ababa University, CURE Hospital in Addis Ababa, Ethiopia, and the University of Toronto. This study is reported in accordance with the Strengthening the Reporting of Observational Studies in Epidemiology (STROBE) reporting guideline and International Society for Pharmacoeconomics and Outcomes Research (ISPOR) reporting guideline.^21,22^ This study was conducted in Addis Ababa, Ethiopia, between July 1, 2019, and January 30, 2020. All participants provided informed consent to participate.

### Study Design and Participants

#### Patient-Proxy Participants

Proxies were necessary because most patients were between 0 and 4 years old and could not reliably self-report.^23^ Only utilities from patient proxies were reported in this study. Patient proxies were recruited from the only 2 multidisciplinary cleft hospitals in Ethiopia, which are both located in Addis Ababa. Eligible patient proxies were caregivers for children younger than 18 years with CL/P who were untreated or treated with primary surgical repair. Participants were excluded if their children had cleft-associated syndromes or mild disease forms, such as forme fruste or bifid uvula. Those who did not understand English or Amharic, the official language of Ethiopia, or were incapable of providing informed consent were excluded. Patient proxies were asked to imagine they were their child in completing the utility assessments and were not given any vignettes or pictures.

#### Societal Participants

Recruitment and selection of societal participants was conducted by convenience sampling in Addis Ababa. Convenience sampling is a nonprobability sampling method that relies on data collection from population members who are conveniently available to participate based on the location of our research associate. Our research associate approached and recruited participants directly. Participants were recruited in coffee shops, around the workplace, and around the home of the research associate. Eligible participants were 18 years and older with no personal or family history of CL/P. The national distribution of males-to-females was targeted. Those who did not understand Amharic or English or were incapable of providing informed consent were excluded.

Societal participants were randomized to receive vignettes for 1 cleft type (cleft lip [CL], cleft palate [CP], or combined CL/P) and measured utilities for both untreated and treated vignettes (eAppendix 1 in the Supplement). Treatment referred to primary surgical repair. Vignettes were created using the World Health Organization definition of health^10^ and further developed with clinical and cultural input from the multidisciplinary cleft teams at Addis Ababa University and the University of Toronto. Male and female CL/P pictures were used to account for sex differences.

### Demographic Survey and Utility-Based HRQOL

A demographic questionnaire elicited information regarding age, religion, ethnicity, occupation, marital status, education, and geographic region of residence, because these are social determinants of health that be associated with the utility value. Utilities were represented on an interval scale from 0 (death) to 1 (perfect health), where a higher value represents better HRQOL.^9^ Utilities can be elicited using indirect and direct measurements. Indirect utility measurements use pre-existing questionnaires (eg, EuroQol-5D [EQ-5D]) that do not capture the impact of facial appearance, speech deficiencies and oral health on HRQOL, and may underestimate CL/P disease burden.^24^ We chose to use direct utility measurements in our study as they capture all dimensions by assessing overall HRQOL in relation to perfect health and death.^9,23^

Three different direct utility measurements: visual analog scale (VAS), time trade-off (TTO) and standard gamble (SG), were used to measure HRQOL (eAppendix 1 in the Supplement).^9^ VAS measures health on a scale from 0 (immediate death) to 100 (perfect health).^9^ TTO assesses the choice between a fixed number of years lived with the disease (eg, 30 years) that a person may be willing to trade for a number of years in perfect health.^9^ SG assesses the choice associated with taking an imaginary pill with a specified risk of either immediate death or perfect health or not taking the pill and remaining in the same health state.^9^

Forward and backward translation of the utility assessments and the vignettes from English to Amharic were conducted by 2 independent Ethiopian translators. Translation was reviewed by a third party and discrepancies were discussed. Ethiopian research associates fluent in Amharic and English were then trained to conduct standardized in-person interviews by the principle investigator (K.Y.C.) and a methods expert (G.B.G.) with previous experience supervising utility studies in Ethiopia. To ensure the comprehensibility of the utility assessments and the vignettes, a pilot study was conducted on 12 graduate students from Addis Ababa University in Ethiopia.^25^ Feedback was then incorporated before use with the target populations. Standardized in-person interviews were conducted for the demographic questionnaire and direct utility assessments. Data were collected by Ethiopian research associates and verified by the principle investigator (K.Y.C.) during the recruitment period.

### Feedback Survey

Feedback on utilities was elicited from all participants using a 5-point Likert scale because it is easy to conceptualize and previously used in other LMICs.^26^ The 5-point scale measures participant understanding, where 1 represents no understanding, and 5 represents complete understanding.

### Sample Size

A sample of 44 participants per group was targeted to detect 95% CIs half-width precision of 0.05. This precision was selected to balance both the uncertainty of the estimate and the feasibility of achieving the targeted sample size. An SD of 0.18 was assumed based on the only existing CL/P utility estimate, which came from a high-income setting.^27^

#### Patient-Proxy Participants

Six patient-proxy groups were included to account for treated and untreated CL, CP, and CL/P, each requiring 44 participants. These groups were targeted for a total sample size of 264 participants.

#### Societal Participants

Three groups of societal participants were needed to complete vignettes for CL, CP, and CL/P. All participants within each group completed 2 vignettes (untreated and treated), where treatment referred to primary surgical repair. The total targeted sample size was 132 participants.

### Statistical Analysis

Descriptive statistics were completed for demographic variables (Table 1). Continuous data were presented as a mean (SD) or median (IQR) as appropriate. Categorical data were reported as frequencies and percentages and compared based on the key predictor of treatment using the χ^2^ test. Statistical significance was set at *P* < .05, and SAS statistical software, version 9.4 (SAS Institute) was used for analysis.

**Table 1.** Demographic Characteristics of Patient-Proxy Participants

Variable	Cleft lip (N = 96)				Cleft palate (N = 40)				Cleft lip and palate (N = 167)			
	Treated (N = 24)	Untreated (N = 72)	*P* value	Missing	Treated (N = 16)	Untreated (N = 24)	*P* value	Missing	Treated (N = 89)	Untreated (N = 78)	*P* value	Missing
Patient age, No. (%)												
<18 mo	9 (38)	69 (96)	<.001	NA	0	18 (75)	<.001	NA	11 (12)	70 (90)	<.001	NA
≥18 mo	15 (63)	3 (4)		NA	16 (100)	6 (25)		NA	78 (88)	8 (10)		NA
Mean (SD), y	6.2 (4.7)	2.7 (0.6)		NA	5.5 (2.6)	4.3 (2.5)		NA	6.4 (3.8)	3.7 (2.3)		NA
Patient sex, No. (%)												
Male	11 (46)	28 (39)	.63	NA	12 (50)	6 (37.5)	.53	NA	52 (59)	43 (55)	.64	NA
Female	13 (54)	44 (61)		NA	12 (50)	10 (62.5)		NA	36 (41)	35 (45)		NA
Proxy sex, No. (%)												
Male	15 (62.5)	26 (36)	.03	NA	7 (44)	9 (37.5)	.75	NA	26 (29)	33 (42)	.10	NA
Female	9 (37.5)	46 (64)			9 (56)	15 (62.5)			63 (71)	45 (58)		
Location outside of Addis Ababa, No. (%)												
Yes	11 (46)	42 (66.7)	.09	9 (9)	5 (31)	21 (87.5)	<.001	1 (2.5)	34 (43)	48 (71)	<.001	20 (12)
No	13 (54)	21 (33.3)			11 (69)	2 (12.5)			45 (57)	20 (29)		
Christian religion, No. (%)												
Yes	20 (83)	50 (69)	.28	NA	10 (62.5)	16 (66)	>.99	NA	69 (88)	61 (88)	>.99	NA
No	4 (17)	22 (51)		NA	6 (37.5)	8 (33)		NA	20 (22)	17 (22)		NA
Oromo ethnicity, No. (%)												
Yes	5 (25)	26 (38)	.13	7 (7)	4 (40)	9 (45)	>.99	10 (15)	19 (27)	31 (44)	.05	26 (16)
No	15 (75)	43 (62)		NA	6 (60)	11 (55)		NA	51 (73)	40 (54)		NA
Proxy unable to read, No. (%)^a^												
Yes	5 (21)	6 (8)	.14	NA	2 (12.5)	0	.15	NA	13 (15)	14 (18)	.67	NA
No	19 (79)	66 (92)		NA	14 (87.5)	24 (100)		NA	76 (85)	64 (82)		NA
Income, No. (%)^b^												
>National mean	20 (83)	61 (85)	>.99	NA	16 (100)	17 (71)	.03	NA	78 (88)	69 (88)	>.99	NA
≤National mean	4 (17)	11 (15)		NA	0	7 (29)		NA	11 (12)	9 (12)		NA

Abbreviation: NA indicates not applicable.

^a^ Literacy of the proxy was used to assess education level.

^b^ Income refers to mean monthly income.

#### Patient-Proxy Perspective

Mean utilities based on treatment were compared using a 2-tailed independent samples *t* test. A multivariable regression model was then created based on clinical (K.Y.C., C.R.F., A.H., M.E.) and cultural expertise (G.B.G., M.E.) regarding social determinants of health. Variables were also selected based on previous evidence of their association with utility values (eAppendix 1 in the Supplement).^24,28,29,30^ The model assessed the impact of treatment on HRQOL after adjusting for cleft type (CL vs CP or CL/P), patient age (younger vs older or equal to 18 months old), proxy sex (male vs female), proxy ability to read (yes vs no), income (above vs below the national average), religion (Christian vs other), ethnicity (Oromo vs other), and geographical location (Addis Ababa vs other). A list of all ethnic groups that were included in the other category can be found in eAppendix 2 in the Supplement. An ordinary least squares (OLS) regression was used. This model was coupled with a nonparametric bootstrap analysis of 200 samples, given the non-normal distribution of data. The 95% CI limits for parameter estimates were reported.

#### Societal Perspective

Mean utilities based on treatment were compared using paired samples *t* tests. A generalized estimating equation (GEE) analysis clustered by participant assessed the impact of treatment on HRQOL after adjusting for cleft type (CP or combined CL/P vs CL), and key social determinants of health that have previously affected utilities in Ethiopia (ie, sex [male vs female] and income [above vs below the sample average]).^28,29^ The GEE analysis leaves the distribution unspecified, thus a bootstrap analysis was not required. The 95% CI limits for parameter estimates were reported.

#### Missing Data

Missing observations were managed with complete case analysis. If missing observations were more than 5%, multiple imputation was conducted as a sensitivity analysis.^31^

## Results

Overall, 447 participants were assessed for eligibility, of which 312 were patient-proxy participants and 135 were societal participants. Of the 9 patient-proxy participants (3% ) who were excluded, 3 [33%] were excluded because no proxy was present, 3 (33%) because the patient was 18 years or older, 2 (22%) because they refused to complete all 3 utility assessments, and 1 (11%) because the cleft type was not categorized. Five societal participants (4%) were excluded from analysis because of a data collection error. Thus, 303 patient-proxy participants and 130 societal participants were included for final analysis. The patient-proxy participants and the societal participants were similar in age (mean [SD] age, 31.9 [7] years vs 31.7 [10.9] years). The patient-proxy population had a lower mean (SD) monthly income than the societal participants ($153 [$180] USD vs $243 [$178] USD), a lower percentage with postsecondary education (89 [29%] vs 104 [80%]), and a higher percentage of female participants vs male participants (188 [61%] vs 61 [47%]). Patient-proxy demographics were reported in Table 1 and societal demographics are reported in Table 2.

**Table 2.** Demographic Characteristics of Societal Participants

Variable	Cleft lip vignettes (N = 46)	Cleft palate vignettes (N = 40)	Cleft lip and palate vignettes (N = 44)	*P* value	Missing
Age, mean (SD)	29.7 (9.7)	32.8 (11.1)	32.9 (11.9)	.30	NA
Sex, No. (%)					
Male	24 (52)	23 (59)	22 (50)	.78	NA
Female	22 (48)	17 (41)	22 (50)		
Income, No. (%)^a^					
>Mean	19 (41)	13 (33)	16 (36)	.70	NA
≤Mean	27 (59)	27 (67)	28 (64)		
Christian religion, No. (%)					
Yes	37 (80)	37 (92)	40 (91)	.22	NA
No	9 (20)	3 (8)	4 (9)		
Patient proxy unable to read, No. (%)^b^					
Yes	5 (11)	3 (7)	5 (11)	.90	NA
No	41 (89)	37 (93)	39 (89)		
Oromo ethnicity, No. (%)					
Yes	12 (36)	5 (18)	4 (14)	.04	32 (25)
No	21 (64)	27 (82)	29 (86)		
Comorbidities, No. (%)					
Yes	3 (7)	6 (15)	8 (18)	.23	NA
No	43 (93)	34 (85)	36 (82)		

Abbreviation: NA indicates not applicable.

^a^ Income refers to the mean monthly income of the sample.

^b^ This literacy of proxy was used to assess education level.

### Patient-Proxy Participants

For the 303 patient-proxy participants, CL/P was the most common cleft type (167 [54%]), followed by CL (96 [31%]), and CP (40 [13%]). The sample size target was not met by those with treated CL (24 [25%]) and CP (treated, 16 [40%]; untreated, 24 [60%]) (Table 1). Mean unadjusted utilities were generally higher with participants who had received treatment compared with participants who had note received treament; however, some of these differences were not statistically significant (Figure). Mean (SD) utilities ranged from 0.63 (0.17) to 0.93 (0.12) participants who had received treatment vs 0.61 (0.31) to 0.75 (0.26) for participants who had not received treatment, depending on the utility instrument and cleft type.

**Figure.**
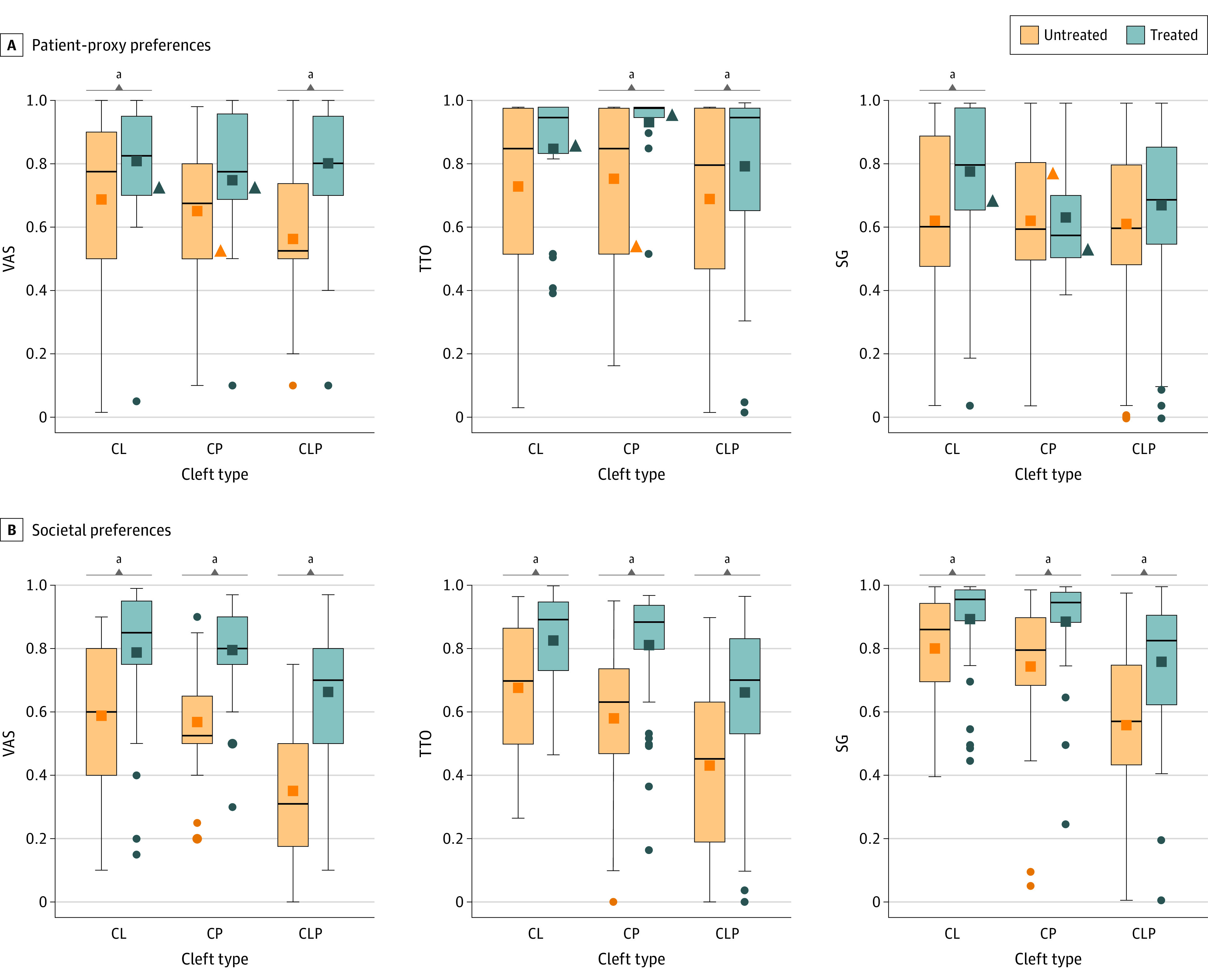
Box Plot Comparison of Mean Utilities Based on Treatment: Independent Samples *t* Test for Patient-Proxy Participants and Paired Sample *t* Test for Societal Participants Bottoms and tops of boxes denote 25th and 75th percentiles, respectively (IQR). Lines within boxes denote medians; squares within boxes denote means; dots denote outliers; and triangles denote samples that did not reach the targeted sample size. SG indicates standard gamble; TTO, time trade-off; VAS, visual analog scale. ^a^Signifies *P* < .05.

On multivariable analysis, treatment was associated with an increase in HRQOL as measured by VAS (0.17 [95% CI, 0.09 to 0.26]; *P* < .001) and TTO (0.15 [95% CI, 0.05 to 0.25]; *P* = .005). Treatment was not associated with a higher SG value (0.08 [95% CI, −0.05 to 0.20]; *P* = .22). With respect to disease severity and cleft type, combined CL/P and CP was associated with a lower HRQOL compared with CL as measured by VAS (Table 3). Social determinants of health that affected utilities from patient proxies were income above the national mean (VAS, 0.10 [95% CI, 0.02 to 0.17]; TTO, 0.11 [95% CI, 0.02 to 0.20]), or religion (Christian vs other: TTO, −0.10 [95% CI, −0.17 to −0.03]). Patient age, proxy sex, education, ethnicity, and geographic location did not significantly affect utility values (Table 3).

**Table 3.** Patient-Proxy Evaluation of the Effect of Treatment for Cleft Lip and/or Palate on Health-Related Quality of Life as Measured by the Visual Analog Scale, the Time Trade-Off, and the Standard Gamble After Adjusting for Health Equity–Related Variables

Variable	Visual Analog Scale (N = 230)		Time trade-off (N = 230)		Standard gamble (N = 228)	
	Parameter estimate (95% CI)	Pr>|t|	Parameter estimate (95% CI)	Pr>|t|	Parameter estimate (95% CI)	Pr>|t|
Intercept	0.62 (0.52 to 0.72)	<.001^a^	0.75 (0.64 to 0.87)	<.001^a^	0.63 (0.49 to 0.77)	<.0001^a^
Treated						
Surgery	0.17 (0.09 to 0.26)	<.001^a^	0.15 (0.05 to 0.25)	.005^a^	0.08 (−0.05 to 0.20)	.22
Untreated	[Reference]		[Reference]		[Reference]	
Cleft type						
Cleft palate	−0.11 (−0.20 to −0.02)	.01^a^	0.05 (−0.05 to 0.16)	.32	0 (−0.12 to 0.12)	.99
Cleft lip	[Reference]		[Reference]		[Reference]	
Cleft type						
Cleft lip and palate	−0.13 (−0.20 to −0.07)	<.001^a^	−0.03 (−0.10 to 0.04)	.48	−0.02 (−0.10 to 0.06)	.64
Cleft lip	[Reference]		[Reference]		[Reference]	
Income^b^						
Above national mean	0.10 (0.02 to 0.17)	.02^a^	0.11 (0.02 to 0.20)	.02^a^	0.03 (−0.07 to 0.14)	.56
Below national mean	[Reference]		[Reference]		[Reference]	
Religion						
Christian	0.02 (−0.05 to 0.08)	.62	−0.1 (−0.17 to −0.03)	.006^a^	0.01 (−0.07 to 0.09)	.84
Other religion	[Reference]		[Reference]		[Reference]	
Proxy sex						
Female	−0.05 (−0.11 to 0.005)	.07	0.02 (−0.05 to 0.09)	.57	0.05 (−0.03 to 0.13)	.2
Male	[Reference]		[Reference]		[Reference]	
Patient age						
≥18 mos	0.01 (−0.07 to 0.10)	.78	−0.07 (−0.17 to 0.03)	.16	−0.02 (−0.14 to 0.10)	.77
<18 mos	[Reference]		[Reference]		[Reference]	
Education						
Proxy unable to read	−0.02 (−0.11 to 0.07)	.72	0.02 (−0.08 to 0.13)	.67	−0.08 (−0.20 to 0.05)	.21
Other education	[Reference]		[Reference]		[Reference]	
Location						
Living outside of Addis Ababa	0.02 (−0.04 to 0.08)	.56	−0.05 (−0.13 to 0.02)	.15	−0.06 (−0.14 to 0.03)	.18
Living within Addis Ababa	[Reference]		[Reference]		[Reference]	
Ethnicity						
Oromo	0.02 (−0.04 to 0.08)	.42	−0.02 (−0.09 to 0.05)	.5	−0.01 (−0.09 to 0.07)	.78
Not Oromo ethnicity	[Reference]		[Reference]		[Reference]	

^a^ Represents *P* < .05.

^b^ Income refers to monthly income, and mean refers to the mean monthly income of the sample.

Missing data (76 [25%]) were managed with complete case analysis for the multivariable regression, and multiple imputation was conducted as a sensitivity analysis. Parameter estimates and their respective 95% CIs remained consistent with multiple imputation (eAppendix 4 in the Supplement). Results also remained consistent with the nonparametric bootstrap (eAppendix 4 in the Supplement). Finally, the feedback survey showed that 288 of participants (95%) reported complete understanding (5/5) of VAS and TTO, and 286 (94%) reported complete understanding (5/5) of SG.

### Societal Participants

Societal participants who completed the CL, CP, or CL/P vignettes were similar with respect to social determinants of health (Table 1). Mean unadjusted utilities for treated vignettes were all significantly higher than untreated vignettes (Figure). Utilities ranged from 0.64 (0.2) to 0.9 (0.15) for participants who had received treatment vs 0.35 (0.21) to 0.8 (0.18) for participants who had not received treatment, depending on the utility instrument and cleft type.

On multivariable analysis, treatment was associated with an increase in HRQOL as measured by VAS (0.20 [95% CI, 0.16 to 0.26]; *P* < .001), TTO (0.17 [0.13 to 0.22]; *P* < .001) and SG (0.11 [95% CI, 0.06 to 0.15]; *P* < .001). With respect to cleft type, combined CL/P was associated with a significant decrease in HRQOL compared with CL or CP as measured by VAS, TTO and SG (Table 4). Being female and treated was associated with a significantly lower HRQOL compared with being male and treated as measured by TTO (−0.05 [95% CI, −0.10 to −0.01]; *P* = .01). Income did not affect utilities (Table 4). There was no missing data for the multivariable regression. Finally, the feedback survey showed that 129 (99%) of participants reported complete understanding (5/5) of VAS and TTO, and 128 (98%) of participants reported complete understanding (5/5) of SG.

**Table 4.** Societal Evaluation of the Effect of Surgical Treatment for Cleft Lip and/or Palate on Health-Related Quality of Life as Measured by the Visual Analog Scale, Time Trade-Off, or Standard Gamble After Adjusting for Cleft Type, Income, and Sex

Variable	Subgroup	Visual Analog Scale (N = 130)		Time trade-off (N = 130)		Standard gamble (N = 130)	
		Estimate (95% CI)	Pr>|Z|	Estimate (95% CI)	Pr>|Z|	Estimate (95% CI)	Pr>|Z|
Intercept		0.60 (0.50 to 0.69)	<.0001^a^	0.70 (0.61 to 0.78)	<.001^a^	0.83 (0.76 to 0.90)	<.001^a^
State	Cleft lip and palate	−0.22(−0.37 to −0.08)	.002^a^	−0.30(−0.44 to −0.15)	<.001^a^	−0.30(−0.44 to −0.16)	<.001^a^
	Cleft palate	−0.03 (−0.15 to 0.09)	.65	−0.10(−0.23 to 0.04)	.15	−0.10 (−0.22 to 0.03)	.12
	Cleft lip	[Reference]		[Reference]	[Reference]		[Reference]
Income^b^	Income > mean	0.02(−0.10 to 0.14)	.71	−0.02(−0.13 to 0.09)	.74	−0.02(−0.11 to 0.08)	.75
	Income < mean	[Reference]		[Reference]	[Reference]		[Reference]
Sex	Female	−0.04(−0.16 to 0.08)	.54	−0.03(−0.14 to 0.08)	.62	−0.05 (−0.14 to 0.04)	.28
	Male	[Reference]		[Reference]	[Reference]		[Reference]
Vignette	Treated (surgery)^c^	0.21(0.16 to 0.26)	<.0001^a^	0.17 (0.13 to 0.22)	<.001^a^	0.11(0.06 to 0.15)	<.001^a^
	Untreated	[Reference]		[Reference]	[Reference]		[Reference]
State*sex	Cleft lip and Palate*Female	0.003(−0.16 to 0.17)	.98	0.02(−0.15 to 0.20)	.80	0.06(−0.10 to 0.22)	.47
	Cleft palate*female	0.05(−0.10 to 0.20)	.55	0.04(−0.13 to 0.20)	.65	0.10(−0.05 to 0.25)	.18
	Cleft lip*female	[Reference]		[Reference]	[Reference]		[Reference]
State*income^b^	Cleft lip and palate* Income>Average	−0.02(−0.19 to 0.14)	.78	0.05(−0.12 to 0.23)	.56	0.07(−0.09 to 0.23)	.38
	Cleft palate*income > average^b^	−0.04(−0.20 to 0.12)	.66	−0.04(−0.21 to 0.13)	.66	−0.05(−0.21 to 0.11)	.56
	Cleft lip* income > average	[Reference]		[Reference]	[Reference]		[Reference]
State*vignette	Cleft Lip and Palate*Treated^c^	0.09(0.04 to 0.15)	.001^a^	0.08(0.03 to 0.13)	.0009^a^	0.10(0.04 to 0.16)	.0004^a^
	Cleft palate*treated^c^	0.03(−0.02 to 0.07)	.29	0.07(0.02 to 0.12)	.005^a^	0.05(0.005 to 0.10)	.03^a^
	Cleft lip*treated	[Reference]		[Reference]	[Reference]		[Reference]
Vignette*income	Treated *Income>Average^b^^,^^c^	−0.001(−0.04 to 0.04)	.98	0.03(−0.02 to 0.07)	.24	0.01(−0.03 to 0.06)	.57
	Treated*income < average	[Reference]		[Reference]	[Reference]		[Reference]
Vignette*sex	Treated*female^c^	−0.03(−0.07 to 0.01)	.20	−0.05(−0.10 to −0.01)	.01^a^	−0.04(−0.08 to 0.01)	.14
	Treated*male	[Reference]		[Reference]	[Reference]		[Reference]

Abbreviation: * indicates an interaction between 2 variables.

^a^ Represents *P* < .05.

^b^ Income refers to monthly income, and mean refers to the mean monthly income of the sample. The sample mean was selected over the national mean because the majority of societal participants had a higher income than the national mean.

^c^ Treated refers to the vignette with primary surgical repair.

## Discussion

To our knowledge, this the first study that elicits utilities for a surgical disease from the patient and societal perspective in an LMIC. Surgical treatment, income, religion, and sex were all significantly associated with utility-based HRQOL, depending on the utility instrument and the participant perspective.

Mean utility values for untreated CL/P in Ethiopia are almost half the respective utilities reported in a high-income setting, such as Canada.^27^ This reveals a significantly greater severe disease burden than was previously reported for CL/P. The significant disparity in HRQOL in LMICs compared with high-income settings have also been reported for other diseases, such as utilities for a minor or major AIDS defining illness in Uganda compared with Canada.^32,33^ The contrast in HRQOL for the same disease in LMICs compared with high-income settings emphasizes the need for context-specific HRQOL measures to inform CEAs and resource allocation decisions in LMICs.

Current CL/P CEAs for LMICs use disability weights (DWs) from the Global Burden of Disease (GBD) studies.^13,34^ DWs from the GBD study are alternative measures of HRQOL, which are reported as being universal but are largely derived from high-income settings, lack consideration for local social determinants of health, and omit the patient perspective.^18,19^ DWs are reported on a similar interval scale as utilities; however, 0 represents perfect health and 1 represents death on the DW scale. The GBD studies suggest that the disease severity of CL/P (DW, 0.011 to 0.115) is comparable to mild alopecia (DW, 0.011) or moderate neck pain (DW, 0.114), which starkly contrasts with the HRQOL reported from Ethiopian participants in our study.^18^ Furthermore, the incremental benefit associated with surgical treatment in our study is more than 3 times higher than the incremental benefit in HRQOL seen in the GBD studies.^20^ Thus, GBD DWs are not universal because they grossly undervalued disease severity and impact of treatment. This may be because GBD descriptions were not context-specific and participants were likely unfamiliar with the severe impact that CL/P can have on HRQOL in LMICs.^5,6,18^ Our study captured these aspects to better represent CL/P health-related quality of life measures in LMIC.

An additional strength of this study is the comprehensive, evidence-based, and culturally sensitive research methodology which increases the comparability of our results and its relevance to LMICs. Eliciting all 3 direct utility measurements facilitates the standardized comparison of CL/P to any study using VAS, TTO, or SG reported from the patient or societal perspective. Our study found that the utility for treated CL/P is still lower than the average utility for the general Ethiopian population.^24^ Thus, there is an opportunity to consider utilities for multidisciplinary management. Primary surgical CL/P repair is the most common form of treatment seen in LMICs; however, it contrasts starkly from the complex, longitudinal and multidisciplinary standard of care for CL/P in high-income settings.

Primary surgical repair improves HRQOL after adjusting for social determinants of health, except when measured by SG from patient proxies. The lack of correlation between SG and other measurements has been reported in other patient or proxy studies; however, the reasons are unclear.^35,36,37^ SG is the only utility instrument that incorporates risk, which is a factor intrinsic to decisions made regarding health. Proxies in LMICs may be more risk tolerant because of cultural stigma, difficulties accessing care, and exposure to a relatively higher infant or child mortality compared with high-income settings. Caregiver burden was not evaluated, but this may also affect risk tolerance and proxy perception of SG.^35^ Furthermore, there are concerns that SG is challenging to understand.^9,23^ It is a limitation that the surveys were pilot tested with students instead of patient proxies or societal participants. In our study, 94% of patient proxies reported that they fully understood SG; however, this may not reflect their actual level of understanding. Eliciting details on caregiver burden, risk tolerance, and interviewer perception of patient proxy understanding in future studies could help explain the lack of correlation between SG and other measurements.

### Limitations

This study had limitations. These include the cross-sectional design, the limited sample of children with CP and treated CL, and the selection bias toward those who live in Addis Ababa, the capital city of Ethiopia. Our results could be further explored in a prospective longitudinal study to mitigate between-patient variability from the patient-proxy participants. In this study, income in the CP population was the only variable that differed between treated and untreated patient proxies and affected utility-based HRQOL. Interestingly, untreated patients with CP had a higher income compared with treated patients. Since a higher income was associated with better HRQOL, our study may still have overestimated utilities for untreated CP. Additionally, a longer recruitment period may capture more patients for the CP and treated CL group. Failure to meet the target sample sizes in these groups may contribute to why differences between the mean treated vs untreated utilities in these groups were not significant. Finally, there is a selection bias toward those in Addis Ababa, which may not be fully represent the population in Ethiopia. With respect to patients and their proxies, the majority of patients did live in Addis Ababa, however rural patients across the country who had access to non-governmental support for travel and accommodation were also recruited in this study. With respect to societal participants, societal participants were only recruited from Addis Ababa where participants have a higher education and have a higher income than the national mean; however, we were able to target the national distribution of males to females. Future utility studies targeting rural participants in Ethiopia would better represent the association of income, geography, and education with HRQOL.

This study aligns with recent World Health Assembly resolutions urging member states to establish surgical care as a component of universal health coverage, to strengthen capacity for health technology assessments to inform policy decisions, and to accelerate efforts addressing social determinants of health that lead to unequal distribution of health resources.^12,38,39^ Since the pandemic has further constrained resources, it is imperative that context-specific utility studies that consider social determinants of health be used to better represent HRQOL for more equitable resource allocation decisions in LMICs.

## Conclusions

These findings suggest that previous HRQOL estimates have underestimated the burden of disease and impact of treatment of CL/P in LMICs and neglected significant social determinants of health critical for addressing health inequalities. Our findings show that universal estimates of HRQOL cannot be indiscriminately applied to cost-effectiveness analyses for LMICs as it has been done in the past. Conducting high-quality utility studies in LMICs has unique challenges but continuing with the status quo risks further marginalization of surgical diseases in health care resource allocation.

## Notes

Supplement. **eAppendix 1.** Supplemental Methods Vignettes **eAppendix 2.** Supplemental Methods Utilities **eAppendix 3.** List of Included Ethnicities **eAppendix 4.** Supplemental Results
