# Supplementary material for: Patient-Proxy and Societal Perspectives of Quality-of-Life Utilities in Children With Cleft Lip and Palate Managed With Surgical Repair vs No Repair in Ethiopia
Source: JAMA Netw Open. 2022 Jul 14;5(7):e2220900. doi: 10.1001/jamanetworkopen.2022.20900 (PMC9284333; doi:10.1001/jamanetworkopen.2022.20900)
Supplement: Supplement. — eAppendix 1. Supplemental Methods Vignettes eAppendix 2. Supplemental Methods Utilities eAppendix 3. List of Included Ethnicities eAppendix 4. Supplemental Results [file jamanetwopen-e2220900-s001.pdf]

Supplemental Online Content

Chung KY, Gebretekle GB, Howard A, et al. Patient-proxy and societal perspectives of quality-of-life utilities in children with cleft lip and palate managed with surgical repair vs no repair in Ethiopia. *JAMA Netw Open*. 2022;5(7):e2220900. doi:10.1001/jamanetworkopen.2022.20900

eAppendix 1. Supplemental Methods Vignettes
eAppendix 2. Supplemental Methods Utilities
eAppendix 3. List of Included Proxy Ethnicities
eAppendix 4. Supplemental Results

This supplemental material has been provided by the authors to give readers additional information about their work.

© 2022 Chung KY et al. *JAMA Network Open.*

eAppendix 1. Supplemental Methods Vignettes

Female Vignettes

English and Amharic

© 2022 Chung KY et al. *JAMA Network Open.*

Cleft lip (Untreated)

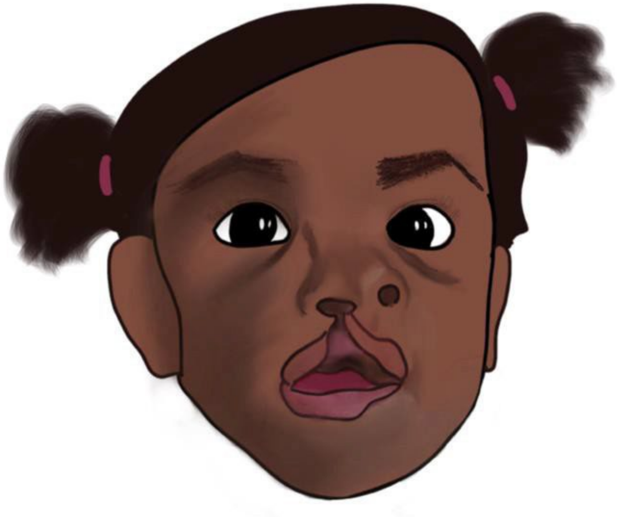

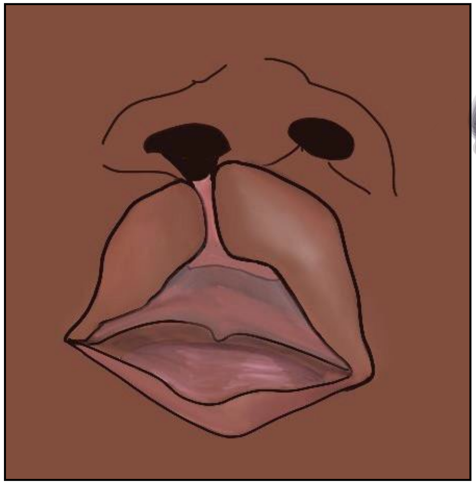

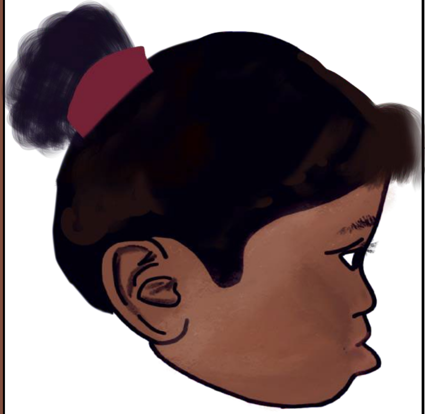

© 2022 Chung KY et al. *JAMA Network Open.*

Assume No Treatment
ህክምና እንደሌለ አስብ/ቢ

Physical:

You look like this.

It’s hard to eat.

You have trouble saying some words.

When your mouth is closed, it looks as though your upper jaw is behind your lower jaw.

Your nose is different.

አካላዊ፦

ይህን ይመስላሉ፡

መብላት ከባድ ነው

አንዳንድ ቃላትን ለመናገር ይከብድዎታል

አፍዎት ሲዘጋ የላይኛው መንጋጋዎት ከታችኛው መንጋጋዎት ኋላ ያለ ይመስላል

አፍንጫዎት የተለየ ነው

Mental:

You are very self-conscious about your mouth, teeth and nose. You are unhappy about your life.

አእምሯዊ:-

የአፍዎ፣ ጥርስዎ እና አፍንጫዎ ሁኔታ በጣም ያሳስብዎታል

በህይዎትዎ ደስተኛ አይደሉም።

Social:

You are rejected by others, and family People do not understand you sometimes. You avoid school or community events.

ማህበራዊ፦

በሌሎች ሰዎች እና በቤተሰብ ይገለላሉ

አንዳንዴ ሰዎች አይረዱዎትም
ከትምህርት እና ማህበራዊ ኩነቶች ራስዎን ያገላሉ

© 2022 Chung KY et al. *JAMA Network Open*.

Cleft lip (Treated)

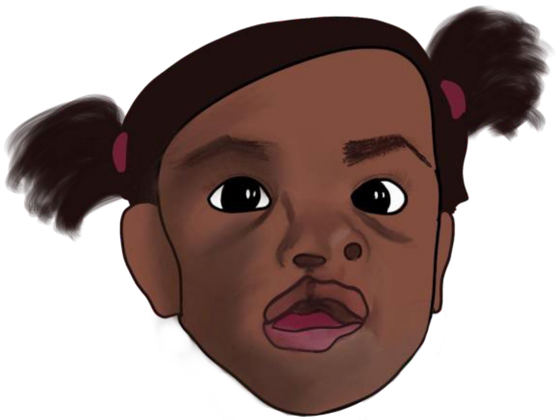

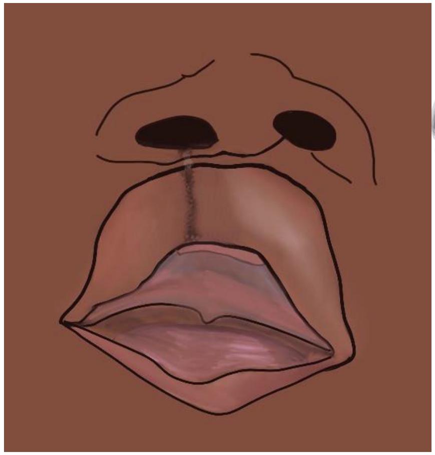

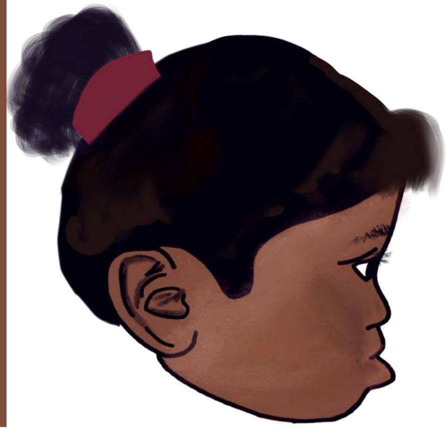

© 2022 Chung KY et al. *JAMA Network Open.*

Physical:

You look like this.

It's easier to talk.

It's easier to eat.

When your mouth is closed, it looks as though your upper jaw is behind your lower jaw.

Your nose is different.

አካላዊ፡-
ይህን ይመስላሉ
ለመናገር ቀለል ያለ ነው
ለመብላት ቀለል ያለ ነው
አፍዎት ሲዘጋ የላይኛው መንጋጋዎት ከታችኛው መንጋጋዎት ኋላ ያለ ይመስላል።
አፍንጫዎት የተለየ ነው

Mental:

You are self-conscious about your scar.

You are happier with your teeth, and your mouth.

አእምሯዊ፡-
የጠባሳዎት ሁኔታ ያሳስብዎታል
በጥርስዎት እና በአፍዎት የተሻለ ደስተኛ ነዎት

Social:

You are more accepted by others and family.

People understand what you say. You feel less bullied. It's easier to make friends. You now go to any social gatherings including school or community events

ማህበራዊ፡-
በሌሎች ሰወች እና በቤተሰብ የተሻለ ተቀባይነት አለዎት
ሰዎች የምትናገረ(ሪ)ውን ይረዱዎታል

ሌሎችን መፍራትዎ ቀንሷል።
ጓደኞችን ማፍራት ቀለል ብሏል
አሁን ትምህርት እና ማህበራዊ
ኩነቶች ጨምሮ በማንኛዉም
ማህበራዊ ስብስቦች ይሳተፋሉ

© 2022 Chung KY et al. *JAMA Network Open.*

Cleft lip and Palate
(Untreated)

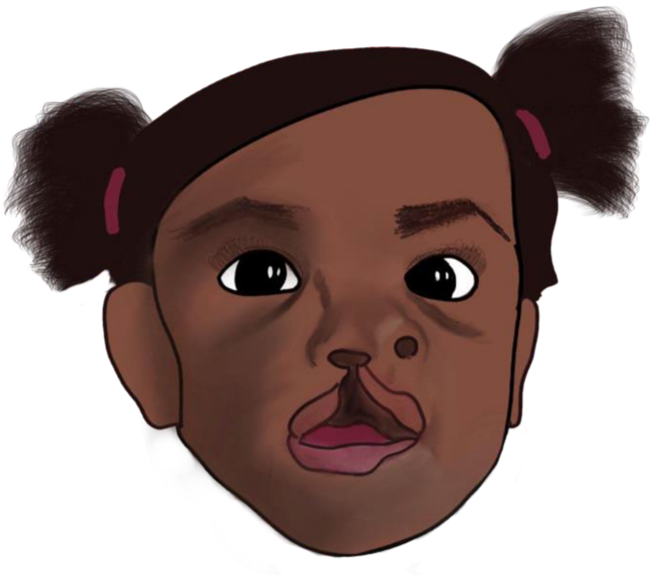

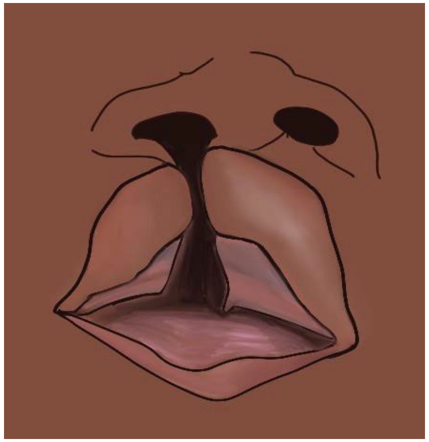

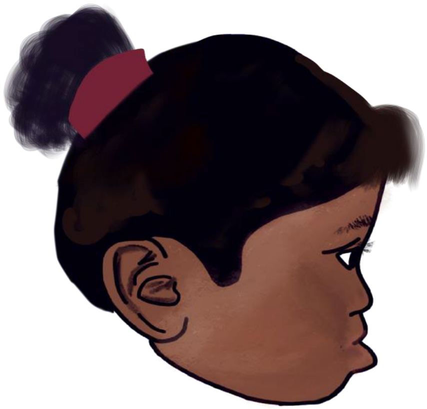

© 2022 Chung KY et al. *JAMA Network Open.*

Assume No Treatment

ህክምና እንደሌለ አስብ/ቢ

Physical: You look like this.

There is a hole connecting your nose and mouth. You have trouble saying words. Food goes into your nose.

You lose weight because you don't eat well. You get ear infections. Your teeth grow in the cleft.

When your mouth is closed, it looks as though your upper jaw is behind your lower jaw. Your nose is different

አካላዊ፡-
ይህን ይመስላሉ፡
አፍንጫዎትን እና አፍዎትን የሚያገናኝ ቀዳዳ አለ።
ቃላትን ለመናገር ይከብድዎታል
ምግብ ከአፍዎ ወደ አፈንጫዎት ይሄዳል
ባግባቡ ስለማይመገቡ ክብደት ይቀንሳሉ
የጆሮ ኢንፌክሽኖች ያጋጥምዎታል
በስንጠቁ መሃል ጥርስዎት ያድጋል
አፍዎ ሲዘጋ የላይኛው መንጋጋዎት ከታችኛው መንጋጋዎት ኋላ ያለ ይመስላል።
አፍንጫዎት የተለየ ነው

Mental:

You are very self-conscious about your mouth, teeth and nose. You are unhappy about your life.

አእምሯዊ:-
የገጽታዎ እና የአነጋገርዎ ሁኔታ በጣም ያሳስብዎታል
በህይዎትዎ ደስተኛ አይደሉም።

Social:

You are rejected by others, and family. **People do not understand you often.** You avoid school or community events.

ማህበራዊ፡-

ም ከትምህርት ወይም ማህበራዊ ኩነቶች ራስዎን ያገላሉ

በሌሎች ህጻናት እና በቤተሰብ ይገለላሉ

ብ
ዙ

ጊ
ዜ

ሰ
ዎ
ች

የ
ም
ት
ና
ገ
ረ
(
ሪ
)
ው
ን

ቃ
ላ
ት

አ
ይ
ረ
ዱ
ዎ
ት

© 2022 Chung KY et al. *JAMA Network Open*.

Cleft lip and Palate (Treated)

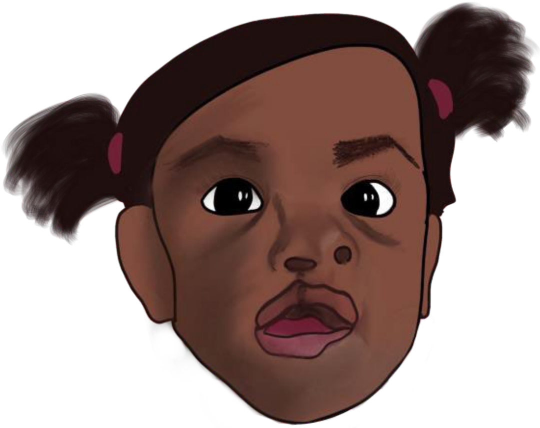

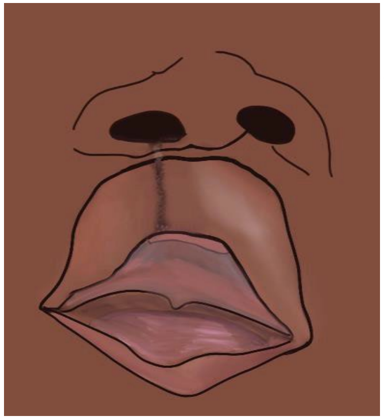

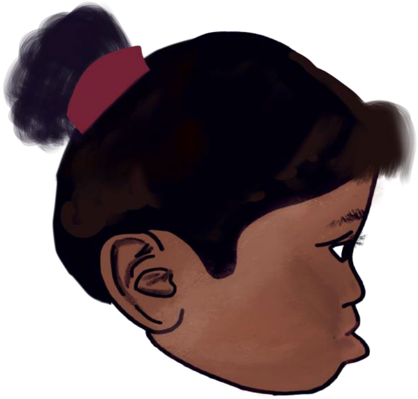

© 2022 Chung KY et al. *JAMA Network Open.*

Physical:

There is no hole between your nose and your mouth.

It is easier to speak, and may be difficult to say some words. Food no longer gets in your nose.

You no longer get frequent ear infections.

You are gaining weight because you are eating well.

Your nose is still lopsided.When your mouth is closed, it still looks as though your upper jaw is behind your lower jaw.

አካላዊ፡-

አፍንጫዎትን እና አፍዎትን የሚያገናኝ ቀዳዳ የለም።
ለመናገር ቀለል ያለ ነው ቢሆንም አንዳንድ ቃላትን ለማለት ሊከብድ ይችላል
ምግብ ከአፍዎት ወደ አፈንጫዎት መግባቱ ቀርቷል
ተደጋጋሚ የጆሮ ኢንፌክሽኖች ማጋጠሙ ቀርቷል
ባግባቡ መመገብ ስለቻሉ ክብደት መጨመር ጀምረዋል
አፍንጫዎት አሁንም የተጣመመ ነው
አፍዎ ሲዘጋ አሁንም የላይኛው መንጋጋዎት ከታችኛው መንጋጋዎት ኋላ ያለ ይመስላል።

Mental:**You are self-conscious about the scar.You are happier with your teeth and your mouth.**

አእምሯዊ:-

የጠባሳው ሁኔታ ያሳስብዎታል
በጥርስዎት እና በአፍዎት የተሻለ ደስተኛ ነዎት

Social:

You get teased less by other kids, family.
People still may not understand what you say, but it is better than before. You attend school or community events.

ማህበራዊ፡-

ሌሎች ህጻናት እና ቤተሰብ ባንተ/ቺ ማሾፋቸውን ቀንሰዋል

አሁንም ሰዎች የምትናገረ(ሪ)ውን ቃላት ላይረዱ ይችላሉ ነገር ግን ከበፊቱ የተሻለ ነው

ትምህርት ወይም ማህበራዊ
ኩነቶች ላይ ይሳተፋሉ

© 2022 Chung KY et al. *JAMA Network Open*.

Cleft Palate Only (Untreated)

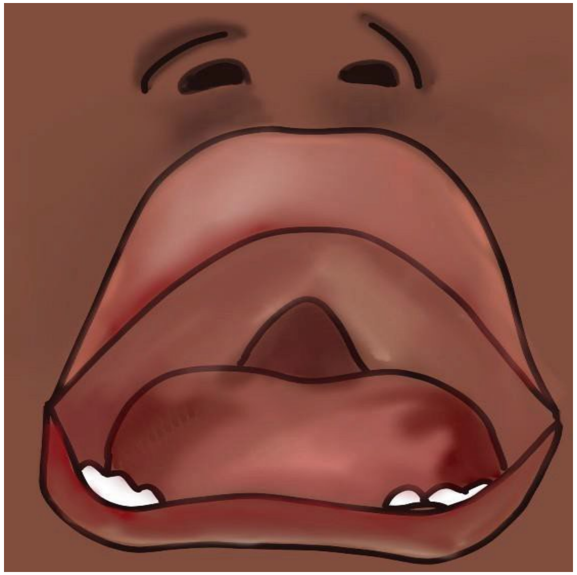

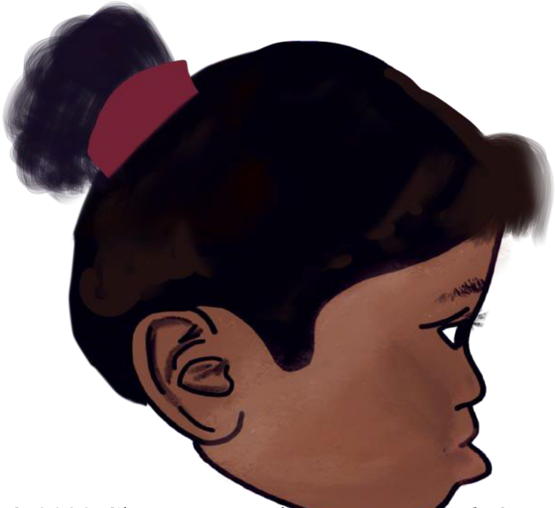

© 2022 Chung KY et al. *JAMA Network Open*.

Assume No Treatment

ህክምና እንደሌለ አስብ/ቢ

Physical:

There is also a hole connecting your nose and your mouth. You have trouble saying some words.

Food goes into your nose.

You lose weight because you don't eat well. You get ear infections.

When your mouth is closed, it looks as though your upper jaw is behind your lower jaw.

አካላዊ፡-

አፍንጫዎትን እና አፍዎትን የሚያገናኝ ቀዳዳ አለ። አንዳንድ ቃላትን ለመናገር ይቸገራሉ

ምግብ ከአፍዎት ወደ አፈንጫዎት ይሄዳል ባግባቡ ስለማይመገቡ ክብደት ይቀንሳሉ የጆሮ ኢንፌክሽኖች ያጋጥምዎታል

አፍዎት ሲዘጋ የላይኛው መንጋጋዎት ከታችኛው መንጋጋዎት ኋላ ያለ ይመስላል።

Mental:

You are self-conscious when you talk. You are unhappy with life.

አእምሯዊ:-

ከሰዎች ጋር ሲያወሩ የአነጋገርዎት ሁኔታ ያሳስብዎታል በህይዎትዎ ደስተኛ አይደሉም።

Social:

You are rejected by others, and family. People do not understand you often. You avoid school or community events.

ማህበራዊ፡-

በሌሎች ህጻናት እና በቤተሰብ ይገለላሉ

ብዙ ጊዜ ሰዎች
የምትናገረ(ሪ)ው
ን ቃላት
አይረዱዎትም
ከትምህርት
ወይም
ማህበራዊ
ኩነቶች ራስዎን
ያገላሉ

© 2022 Chung KY et al. *JAMA Network Open.*

Cleft Palate (Treated)

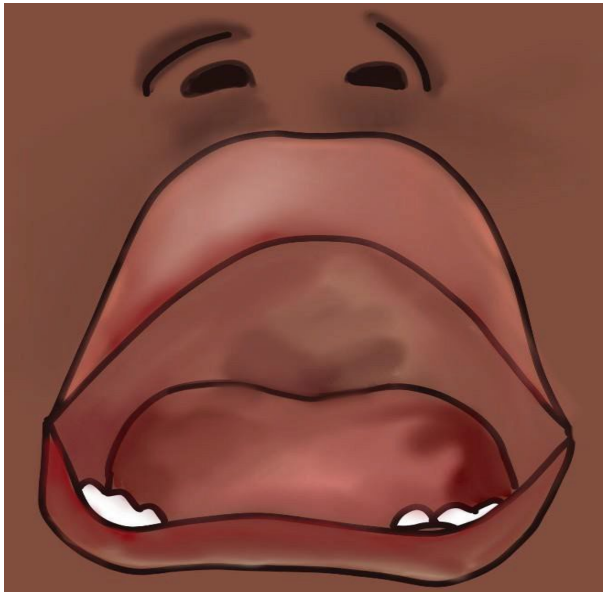

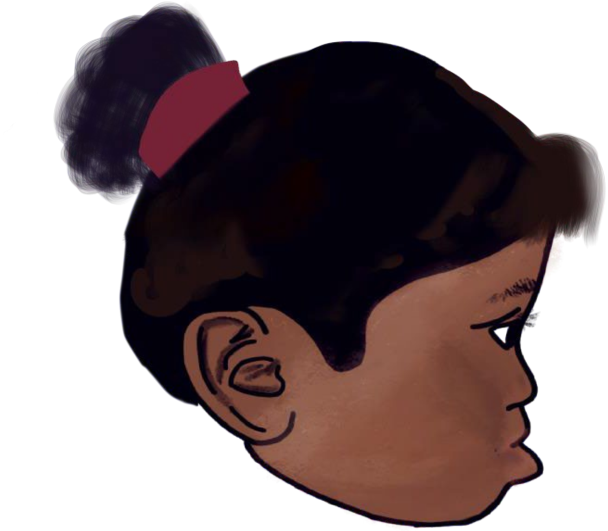

© 2022 Chung KY et al. *JAMA Network Open.*

Physical:

There is no hole between your nose and the roof of your mouth. It is easier to speak, but still difficult to say some words.

Food no longer gets in your nose.

You no longer get frequent ear infections.

You are gaining weight because you are eating well.

When your mouth is closed, it still looks as though your upper jaw is behind your lower jaw.

አካላዊ፡-

በአፍንጫዎት እና በአፍዎት የላይኛው ክፍል መካከል ቀዳዳ የለም

መናገር ቀለል ያለ ነው ግን አሁንም አንዳንድ ቃላትን ማውጣት ከባድ ነው

ምግብ ከአፍዎት ወደ አፈንጫዎት መግባቱ ቀርቷል

ተደጋጋሚ የጆሮ ኢንፌክሽኖች ማጋጠሙ ቀርቷል

ባግባቡ መመገብ ስለቻሉ ክብደት መጨመር ጀምረዋል

አፍዎት ሲዘጋ አሁንም የላይኛው መንጋጋዎት ከታችኛው መንጋጋዎት ኋላ ያለ ይመስላል።

Mental:

You are less self-conscious about the way you talk.

You are happy about the fact there is no hole, and eating no longer makes you worry.

አእምሯዊ:-

ከሰዎች ጋር ሲነጋገሩ ስለአነጋገርዎት ሁኔታ ማሰብዎት ቀንሷል

ቀዳዳ አለመኖሩ እና መመገብ የሚያስጨንቅዎት ሁኔታ በመቅረቱ ደስተኛ ነዎት።

Social:

You are more accepted by others, including family.

People still may not understand what you say, **but it is better than before.**

You now return to school or community events.

ማህበራዊ፡-

በሌሎች ሰዎች እና በቤተሰብ የተሻለ ተቀባይነት አለዎት

አሁንም ሰዎች የምትናገረ(ሪ)ውን ቃላት ላይረዱ ይችላሉ ነገር ግን ከበፊቱ የተሻለ ነው አሁን ወደ ትምህርት ወይም

ማህበራዊ ኩነቶች ተመልሰዋል

© 2022 Chung KY et al. *JAMA Network Open*.

Male Vignettes

Amharic
(English translation similar to Female Version)

© 2022 Chung KY et al. *JAMA Network Open.*

Cleft lip (Untreated)

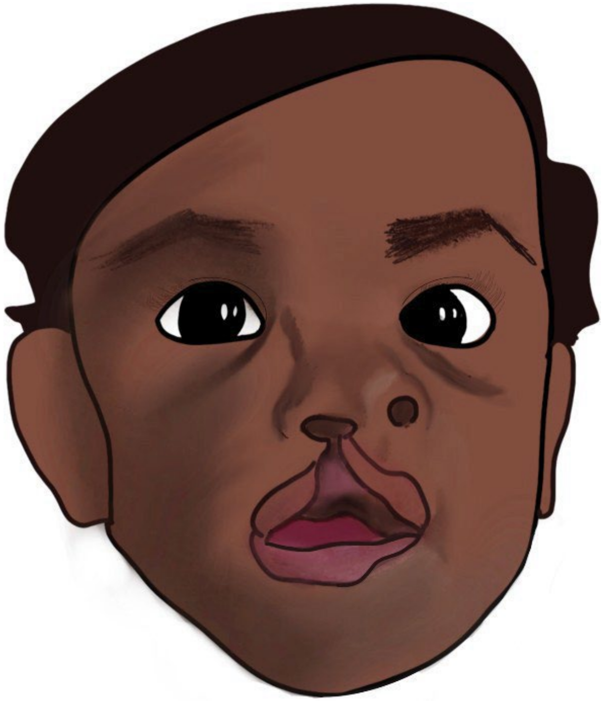

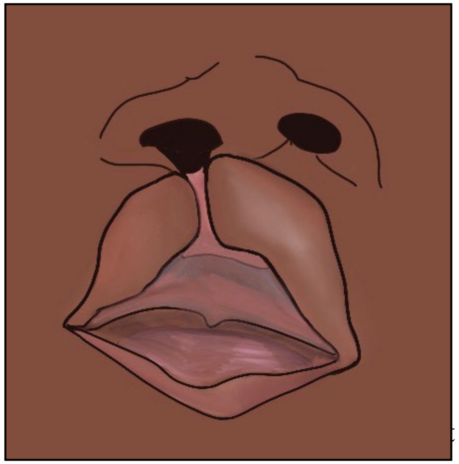

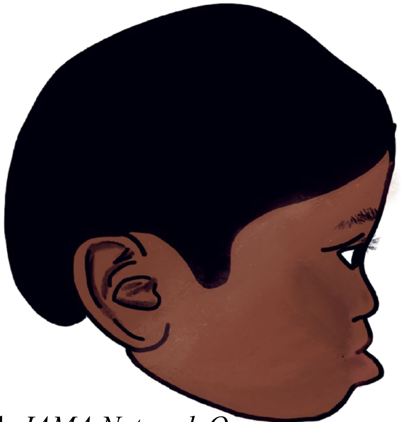

[illegible] al. *JAMA Network Open.*

ህክምና እንደሌለ አስብ/ቢ

አካላዊ፡-

ይህን ይመስላሉ፡

መብላት ከባድ ነው

አንዳንድ ቃላትን ለመናገር ይከብድዎታል

አፍዎት ሲዘጋ የላይኛው መንጋጋዎት ከታችኛው መንጋጋዎት ኋላ ያለ ይመስላል

አፍንጫዎት የተለየ ነው

አእምሯዊ፡-

የአፍዎ፣ ጥርስዎ እና አፍንጫዎ ሁኔታ በጣም ያሳስብዎታል

በህይዎትዎ ደስተኛ አይደሉም።

ማህበራዊ፡-

በሌሎች ሰወች እና በቤተሰብ ይገለላሉ

አንዳንዴ ሰዎች አይረዱዎትም

ከትምህርት እና ማህበራዊ ኩነቶች ራስዎን ያገላሉ

Cleft lip (Treated)

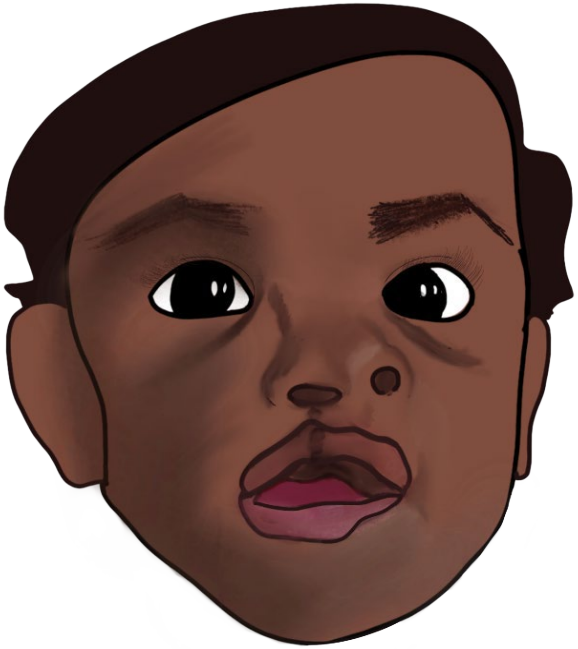

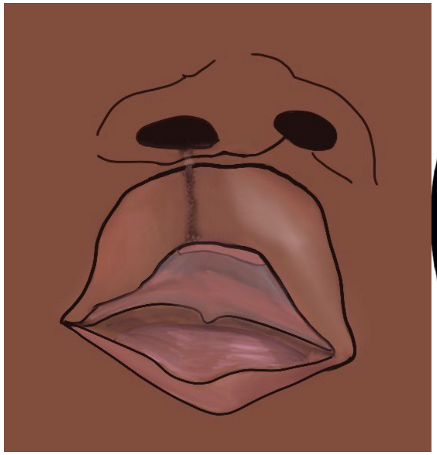

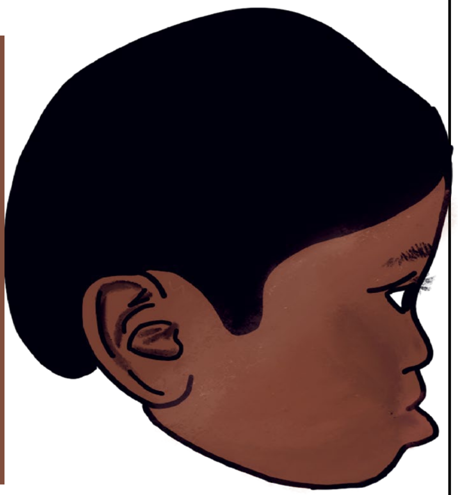

© 2022 Chung KY et al. *JAMA Network Open.*

አካላዊ፡-

ይህን ይመስላሉ
ለመናገር ቀለል ያለ ነው
ለመብላት ቀለል ያለ ነው
አፍዎት ሲዘጋ የላይኛው መንጋጋዎት ከታችኛው መንጋጋዎት ኋላ ያለ ይመስላል።
አፍንጫዎት የተለየ ነው

አእምሯዊ:-

የጠባሳዎት ሁኔታ ያሳስብዎታል
በጥርስዎት እና በአፍዎት የተሻለ ደስተኛ ነዎት

ማህበራዊ፡-

በሌሎች ሰወች እና በቤተሰብ የተሻለ ተቀባይነት አለዎት
ሰዎች የምትናገረ(ሪ)ውን ይረዱዎታል
ሌሎችን መፍራትዎ ቀንሷል። ጓደኞችን ማፍራት ቀለል ብሏል
አሁን ትምህርት እና ማህበራዊ ኩነቶች ጨምሮ በማንኛዉም ማህበራዊ ስብስቦች ይሳተፋሉ

Cleft lip and Palate (Untreated)

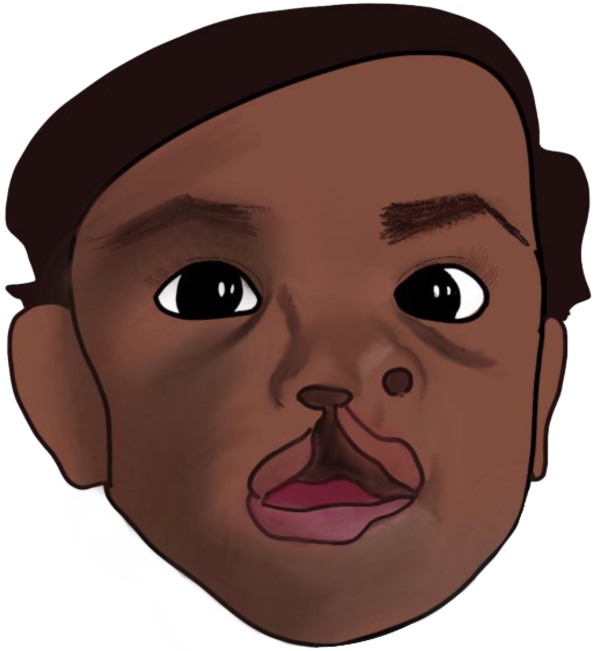

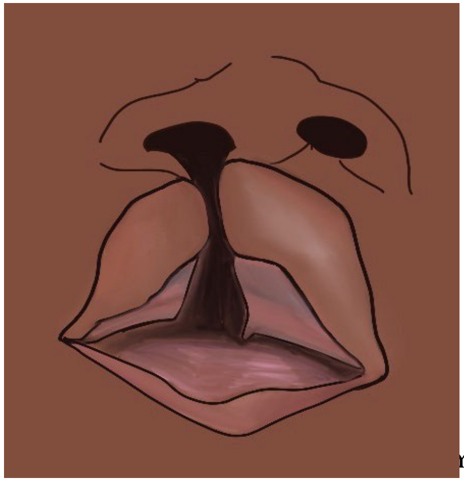

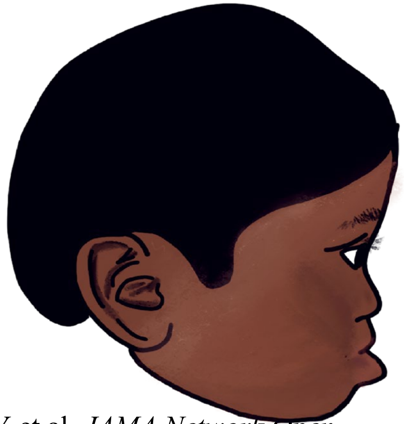

Y et al. *JAMA Network Open.*

ህክምና እንደሌለ አስብ/ቢ

አካላዊ፡-
ይህን ይመስላሉ፡
አፍንጫዎትን እና አፍዎትን የሚያገናኝ ቀዳዳ አለ።
ቃላትን ለመናገር ይከብድዎታል
ምግብ ከአፍዎ ወደ አፈንጫዎት ይሄዳል
ባግባቡ ስለማይመገቡ ክብደት ይቀንሳሉ
የጆሮ ኢንፌክሽኖች ያጋጥምዎታል
በስንጠቁ መሃል ጥርስዎት ያድጋል
አፍዎ ሲዘጋ የላይኛው መንጋጋዎት ከታችኛው መንጋጋዎት ኋላ ያለ ይመስላል።
አፍንጫዎት የተለየ ነው

አእምሯዊ፡-
የገጽታዎ እና የአነጋገርዎ ሁኔታ በጣም ያሳስብዎታል
በህይዎትዎ ደስተኛ አይደሉም።

ማህበራዊ፡-
በሌሎች ህጻናት እና በቤተሰብ ይገለላሉ
ብዙ ጊዜ ሰዎች የምትናገረ(ሪ)ውን ቃላት አይረዱዎትም
ከትምህርት ወይም ማህበራዊ ኩነቶች ራስዎን ያገላሉ

Cleft lip and Palate (Treated)

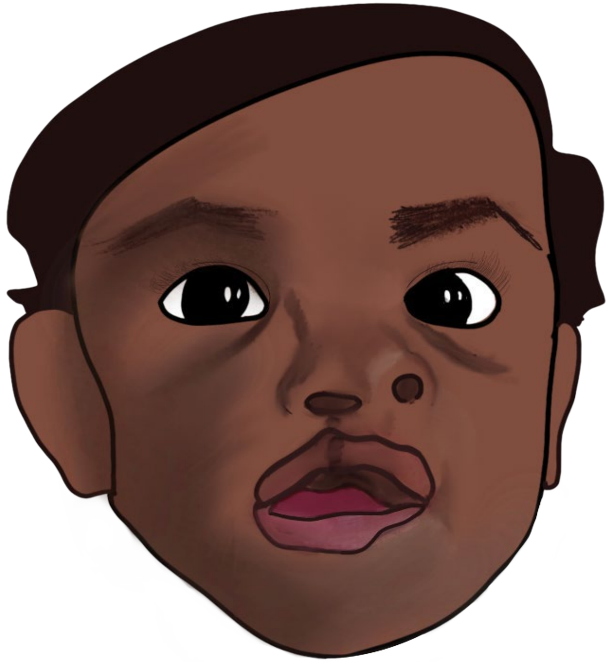

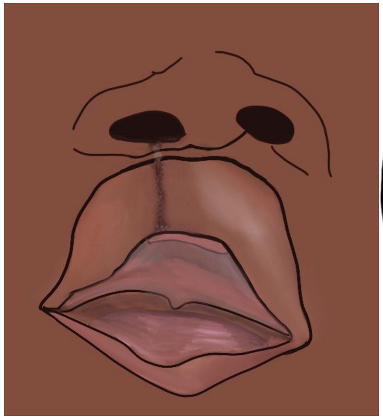

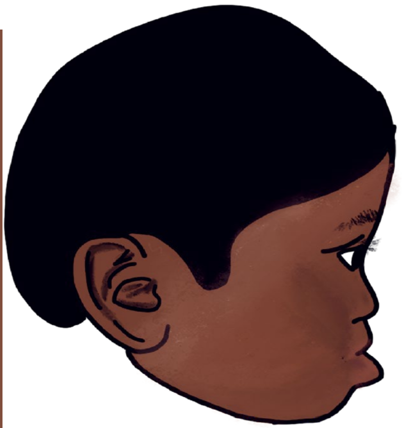

© 2022 Chung KY et al. *JAMA Network Open.*

አካላዊ:-

አፍንጫዎትን እና አፍዎትን የሚያገናኝ ቀዳዳ የለም።

ለመናገር ቀለል ያለ ነው ቢሆንም አንዳንድ ቃላትን ለማለት ሊከብድ ይችላል

ምግብ ከአፍዎት ወደ አፈንጫዎት መግባቱ ቀርቷል

ተደጋጋሚ የጆሮ ኢንፌክሽኖች ማጋጠሙ ቀርቷል

ባግባቡ መመገብ ስለቻሉ ክብደት መጨመር ጀምረዋል

አፍንጫዎት አሁንም የተጣመመ ነው

አፍዎ ሲዘጋ አሁንም የላይኛው መንጋጋዎት ከታችኛው መንጋጋዎት ኋላ ያለ ይመስላል።

አእምሯዊ:-

የጠባሳው ሁኔታ ያሳስብዎታል

በጥርስዎት እና በአፍዎት የተሻለ ደስተኛ ነዎት

ማህበራዊ:-

ሌሎች ህጻናት እና ቤተሰብ ባንተ/ቺ ማሾፋቸውን ቀንሰዋል

አሁንም ሰዎች የምትናገረ(ሪ)ውን ቃላት ላይረዱ ይችላሉ

ነገር ግን ከበፊቱ የተሻለ ነው

ትምህርት ወይም ማህበራዊ ኩነቶች ላይ ይሳተፋሉ

7 - Cleft Palate Only (Untreated)

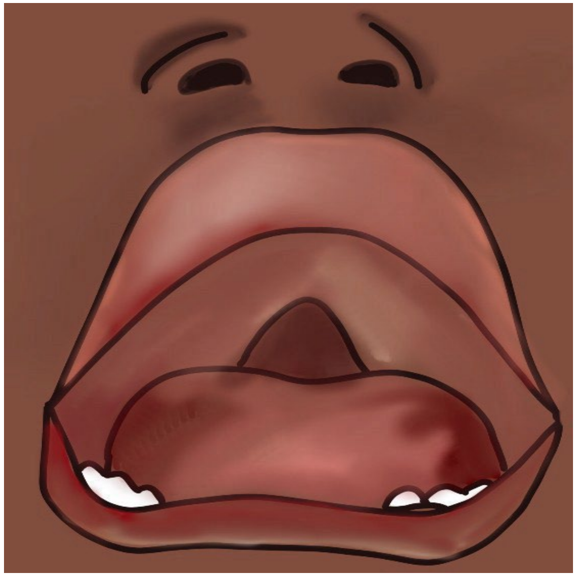

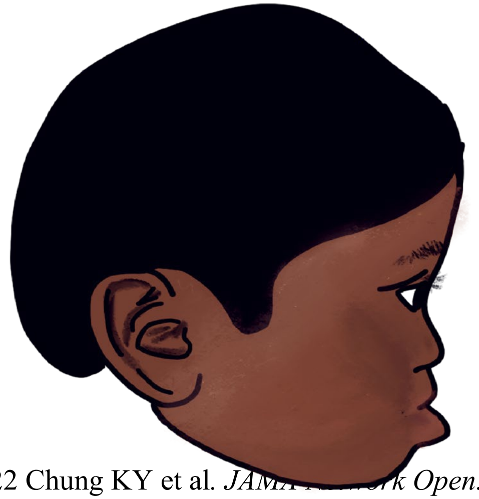

© 2022 Chung KY et al. *JAMA* [illegible]*rk Open.*

ህክምና እንደሌለ አስብ/ቢ

አካላዊ፡-

አፍንጫዎትን እና አፍዎትን የሚያገናኝ ቀዳዳ አለ።
አንዳንድ ቃላትን ለመናገር ይቸገራሉ
ምግብ ከአፍዎት ወደ አፈንጫዎት ይሄዳል
ባግባቡ ስለማይመገቡ ክብደት ይቀንሳሉ
የጆሮ ኢንፌክሽኖች ያጋጥምዎታል
አፍዎት ሲዘጋ የላይኛው መንጋጋዎት ከታችኛው መንጋጋዎት ኋላ ያለ ይመስላል።

አእምሯዊ፡-

ከሰዎች ጋር ሲያወሩ የአነጋገርዎት ሁኔታ ያሳስብዎታል
በህይወትዎ ደስተኛ አይደሉም።

ማህበራዊ፡-

በሌሎች ህጻናት እና በቤተሰብ ይገለላሉ
ብዙ ጊዜ ሰዎች የምትናገረ(ሪ)ውን ቃላት አይረዱዎትም
ከትምህርት ወይም ማህበራዊ ኩነቶች ራስዎን ያገላሉ

8 - Cleft Palate (Treated)

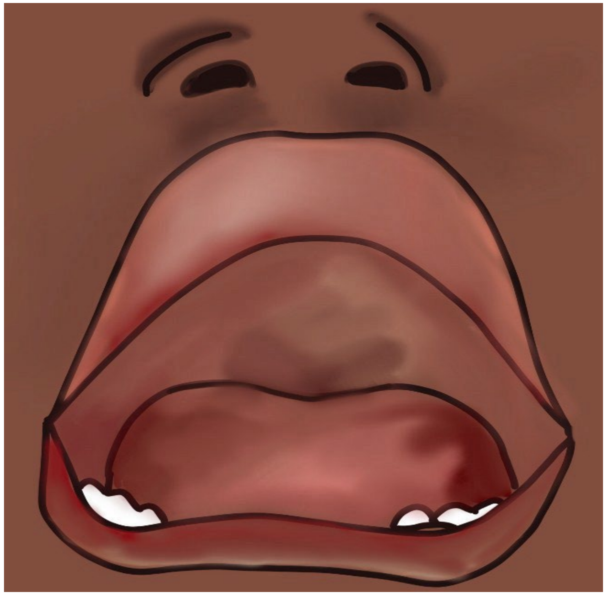

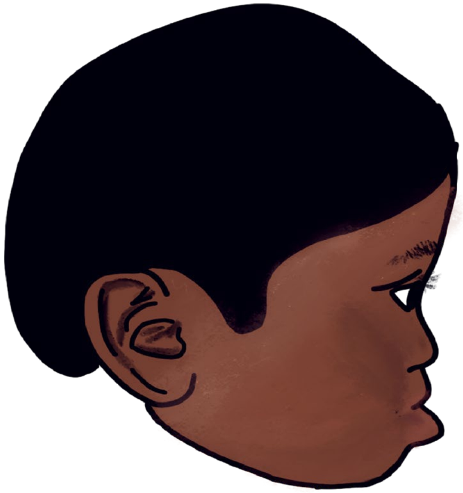

© 2022 Chung KY et al. *JAMA Network Open.*

አካላዊ፡-

በአፍንጫዎት እና በአፍዎት የላይኛው ክፍል መካከል ቀዳዳ የለም

መናገር ቀለል ያለ ነው ግን አሁንም አንዳንድ ቃላትን ማውጣት ከባድ ነው

ምግብ ከአፍዎት ወደ አፈንጫዎት መግባቱ ቀርቷል

ተደጋጋሚ የጆሮ ኢንፌክሽኖች ማጋጠሙ ቀርቷል

ባግባቡ መመገብ ስለቻሉ ክብደት መጨመር ጀምረዋል

አፍዎት ሲዘጋ አሁንም የላይኛው መንጋጋዎት ከታችኛው መንጋጋዎት ኋላ ያለ ይመስላል።

አእምሯዊ፡-

ከሰዎች ጋር ሲነጋገሩ ስለአነጋገርዎት ሁኔታ ማሰብዎት ቀንሷል

ቀዳዳ አለመኖሩ እና መመገብ የሚያስጨንቅዎት ሁኔታ በመቅረቱ ደስተኛ ነዎት።

ማህበራዊ፡-

በሌሎች ሰዎች እና በቤተሰብ የተሻለ ተቀባይነት አለዎት

አሁንም ሰዎች የምትናገረ(ሪ)ውን ቃላት ላይረዱ ይችላሉ

ነገር ግን ከበፊቱ የተሻለ ነው አሁን ወደ ትምህርት ወይም ማህበራዊ ኩነቶች ተመልሰዋል

eAppendix 2. Supplemental Methods Utilities

Visual Analogue Scale

Patient-proxies: Please imagine you are your child

We would like to know how good or bad your health is TODAY. This scale is numbered from 0 to 100. 100 means the best health you can imagine. 0 refers to death Mark an X on the scale to indicate how your health is TODAY. Now, please write the number you marked on the scale in the box below

YOUR HEALTH TODAY =

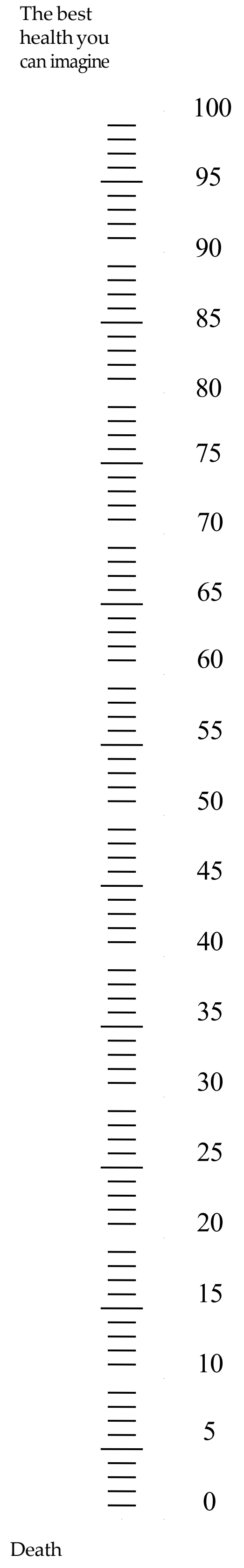

© 2022 Chung KY et al. *JAMA Network Open.*

Visual Analogue Scale English and Amharic

የሚታይ አምሳያ መለኪያ

የህመምተኛው ወኪል፦ እባክዎ ልጅዎትን እንደሆኑ በአይምሮዎ ይሳሉ

የዛሬ የጤና ሁኔታዎ ምን ያህል ጥሩ ወይም መጥፎ እንደሆነ ለማወቅ እንወዳለን
መለኪያው ከ 0 እስከ 100 ድረስ ቁጥሮች አሉት
100 ማለት ሊያስቡት የሚችሉት <u>ከፍተኛው</u> የጤና ሁኔታ ሲሆን 0 ደግሞ ሞትን ያመለክታል።
በመለኪያው ላይ የዛሬ የጤንነትዎ ሁኔታን ለማሳየት የ X ምልክት ያድርጉ
አሁን, በመለኪያው ላይ ምልክት ያደረጉበትን ቁጥር ከስር ባለው ሳጥን ውስጥ ይጻፉ

ዛሬ ያለዎት የጤና ሁኔታ =

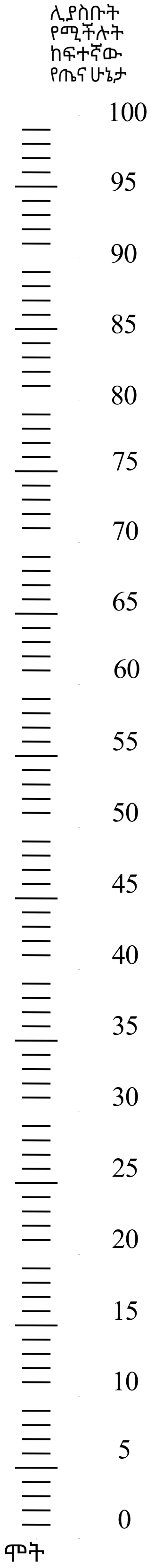

© 2022 Chung KY et al. *JAMA Network Open.*

Time Trade Off

Time Trade-Off Design

Time Trade-Off Design

Researcher:

Start at 30 years of perfect health, then go to 0 years of perfect health. If either choice A or choice B are chosen, continue with 15 years of perfect health. If A is chosen (15 years in perfect health), then go DOWN the number of perfect years by 1 year. If B is chosen (30 years of current health state), then go UP the number of perfect years by 1 year. When choice A and B are the same, you may go into decimal points (starting at 0.5) and changing by increments of 0.1 until choice A and B remain the same. When Choice A and Choice B are the same, the number of years in Choice A over Choice B (30 years) is the utility score. If the participant chooses immediate death (0 years perfect health) over life with cleft lip and/or palate then proceed to “Worse Than Death” Appendix 4B.

Participants:

- ☐ Patient-proxies: Please imagine you are your child. “This health state” refers to the state your child is in right now.
- ☐ Society: Please imagine you are in the health state of your vignette.

Which is better?
Choice A?
Choice B?
Are Choice A and Choice B the same?

Choice A – Years in Perfect Health

1	2	3	4	5	6	7	8	9	10	11	12	13	14	15	16	17	18	19	20	21	22	23	24	25	26	27	28	29	30

Or

Choice B - 30 years where you are right now

1	2	3	4	5	6	7	8	9	10	11	12	13	14	15	16	17	18	19	20	21	22	23	24	25	26	27	28	29	30

Time Trade Off:

Choice A
―――――
30 years

© 2022 Chung KY et al. *JAMA Network Open.*

Time Trade Off Amharic

አየጊዜ ሰጥቶ መቀበል ዲዛይን

የጊዜ ሰጥቶ መቀበል ዲዛይን

ተመራማሪ:-

ከ 30 አመት በፍጹም ጤንነት ጀምር/ሪ እና ወደ 0 አመት በፍጹም ጤንነት ሂድ/ጂ። ምርጫ ሀ ወይም ምርጫ ለ ከተመረጡ ደግሞ ወደ 15 አመት በፍጹም ጤንነት ቀጥል/ይ። ምርጫ ሀ (15 አመት በፍጹም ጤንነት) ከተመረጠ የፍጹም ጤንነት አመታትን በ 1 አመት እየቀነስክ/ሽ ቀጥል/ይ። ምርጫ ለ (30 አመት አሁን ባሉበት ሁኔታ) ከተመረጠ የፍጹም ጤንነት አመታትን በ 1 አመት እየጨመርክ/ሽ ቀጥል/ይ። ምርጫ ሀ እና ምርጫ ለ አንድ አይነት በሚሆኑበት ጊዜ ምርጫ ሀ እና ምርጫ ለ አንድ አይነት ሆነው እስከሚቀጥሉ ድረስ ወደ አስርዮሽ ነጥብ (ከ 0.5 ጀምረህ/ሽ) በመሄድ 0.1 ነጥብ ጨማሪ በማድረግ መቀጠል ትችላለህ/ያልሽ። ምርጫ ሀ እና ምርጫ ለ አንድ አይነት ሲሆኑ፣ በምርጫ ሀ ዉስጥ ያሉት አመታት በምርጫ ለ ውስጥ ባሉት አመታት (30 አመትታ) ሲካፈሉ የሚመጣው ውጤት የዩቲሊቲ ነጥብ ይባላል።

ተሳታፊዎች:

የህመምተኛው ወኪል: ልጅዎትን እንደሆኑ በአይምሮዎ ይሳሉ። “ይህ የጤና ሁኔታ” ልጅዎ አሁን ያለበትን ሁኔታ ያመለክታል።

ማህበረሰብ: በገለጻው ውስጥ ባለው የጤና ሁኔታ እንዳሉ በአይምሮዎ ይሳሉ

የትኛው ይሻላል?
ምርጫ ሀ?
ምርጫ ለ?
ምርጫ ሀ እና ምርጫ ለ አንድ አይነት ናቸው?

ምርጫ ሀ – በፍጹም ጤንነት የሚያልፉ አመታት

1	2	3	4	5	6	7	8	9	10	11	12	13	14	15	16	17	18	19	20	21	22	23	24	25	26	27	28	29	30

ወይም

ምርጫ ለ - **30** አመት አሁን ባሉበት ሁኔታ

1	2	3	4	5	6	7	8	9	10	11	12	13	14	15	16	17	18	19	20	21	22	23	24	25	26	27	28	29	30

የጊዜ ሰጥቶ መቀበል:

ምርጫ ሀ **30**

አመታት

© 2022 Chung KY et al. *JAMA Network Open.*

Standard Gamble Amharic

Standard Gamble:
Lay Public Participant: Pretend you are the story you just read. You have a choice between staying where you are right now current state or taking a gamble (an imaginary drug) with two hypothetical possible outcomes: Chance of perfect health or immediate painless death.

Proxy/Patient: You have a choice between staying where you are right now current state or taking a gamble (an imaginary drug) with two hypothetical possible outcomes: Probability of perfect health or immediate painless death.

For the researcher:

1. If the drug has 100% perfect health (blue) and no risk of death (red) Would you rather stay where you are now or take the drug?

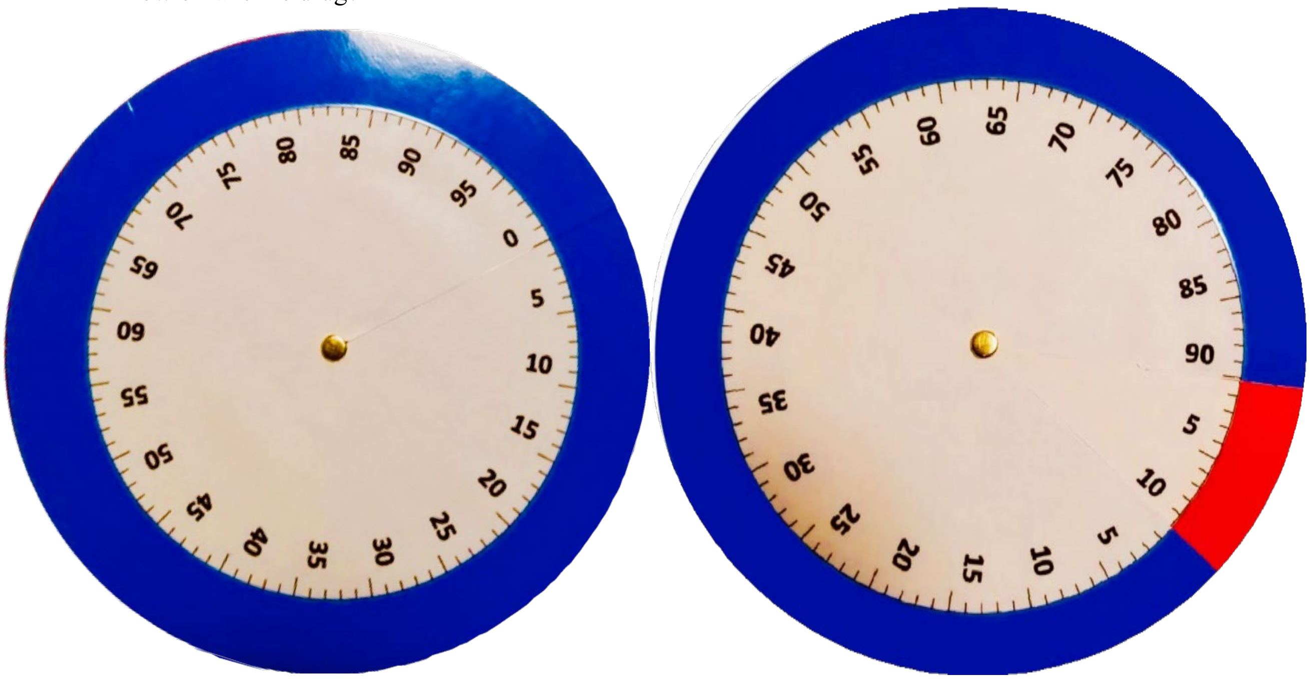

© 2022 Chung KY et al. *JAMA Network Open.*

2. What if the drug had 0% risk of perfect health (blue) and 100% immediate death (red), would you rather stay where you are now or take the drug?

    a. If they choose the drug, proceed to Step 3.

3. What if the drug had a chance of 50% perfect health and 50% immediate death, would you rather stay where you are now or take the drug?
    a. If they choose the drug, proceed to Step 4.
    b. If they choose where they are now, increase to pill 60% perfect health and 40% immediate death.
        - If they are uncertain, go half way: start at 55% perfect health and 45% immediate death
            - if they choose the drug, move to 54% perfect health and 46% immediate death
            - If they choose to stay where they are then the drug will be then 56% perfect health and 44% immediate death
            - And so forth

4. What if the drug had a chance of 40% perfect health and 60% immediate death, would you rather stay where you are now or take the drug?
        - If they are uncertain, go half way: start at 45% perfect health and 55% immediate death
            - if they choose the drug, move to 44% perfect health and 56% immediate death
            - If they choose to stay where they are then the drug will be then 46% perfect health and 54% immediate death
            - And so forth

… and so forth.

Participants are allowed to change their mind. If they are certain, proceed in increments of 10% and if they become uncertain, bounce back and forth as in the example provided in increments of 5% and then 1

© 2022 Chung KY et al. *JAMA Network Open.*

Standard Gamble Amharic

ስታንዳርድ ቁማር:

በህመሙ ቀጥተኛ ተጠቂ ያልሆነ ህብረተሰብ:- አሁን ባነበቡት ታሪክ ውስጥ ያለውን ህመምተኛ እንደሆኑ ያስመስሉ። አሁን ባሉበት ሁኔታ ለመቆየት ወይም ምናባዊ መድሃኒት ለመውሰድ የመቆመር ምርጫ አለዎት። በመቆመር ዉስጥም ምናባዊ መድሃኒቱ ሊከሰቱ የሚችሉ ሁለት መላ ምታዊ ውጤቶች አሉት። ውጤቶቹም ፍጹም ጤንነት የማግኘት እድል ወይም ወዲያውኑ የሚከሰት ህመም አልባ የሆነ ሞት ናቸው።

የህመምተኛው ወኪል / ህመምተኛ:- አሁን ባሉበት ሁኔታ ለመቆየት ወይም ምናባዊ መድሃኒት ለመውሰድ የመቆመር ምርጫ አለዎት። በመቆመር ዉስጥም ምናባዊ መድሃኒቱ ሊከሰቱ የሚችሉ ሁለት መላ ምታዊ ውጤቶች አሉት። ውጤቶቹም ፍጹም ጤንነት የማግኘት እድል ወይም ወዲያውኑ የሚከሰት ህመም አልባ የሆነ ሞት ናቸው።

ለተመራማሪው:

1. መድሃኒቱ 100% ፍጹም ጤንነት (ሰማያዊ) ካለው እና የመሞት ስጋት (ቀይ) ከሌለው አሁን ባሉበት ሁኔታ ለመቆየት ይፈልጋሉ ወይስ መድሃኒቱን ይወስዳሉ?

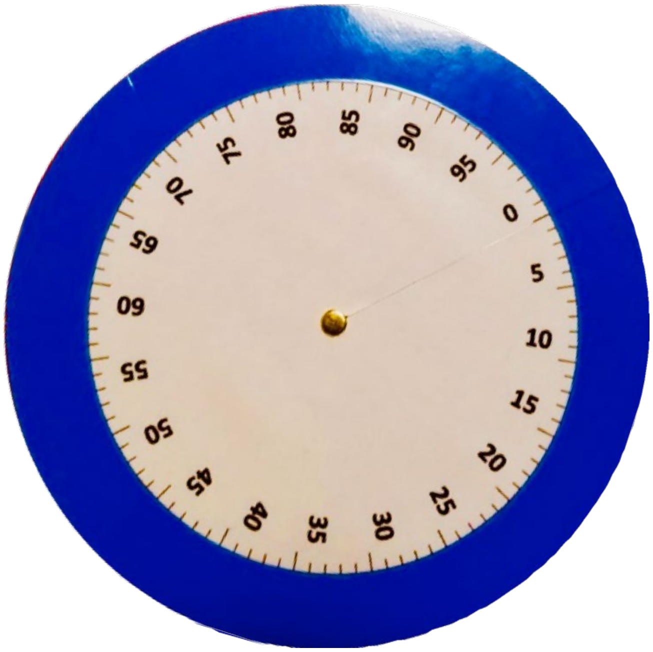

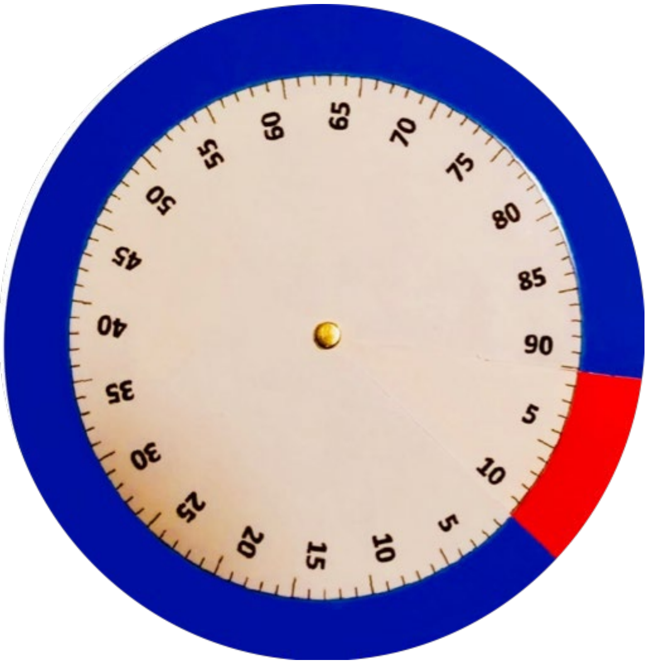

© 2022 Chung KY et al. *JAMA Network Open.*

2. መድሃኒቱ 0% ፍጹም ጤንነት (ሰማያዊ) እና 100% ወዲያውኑ የሚከሰት የመሞት ስጋት (ቀይ) ቢኖረውስ አሁን ባሉበት ሁኔታ ለመቆየት ይፈልጋሉ ወይስ መድሃኒቱን ይወስዳሉ?

    ሀ. መድሃኒቱን ከመረጡ ወደ 3ተኛው ጥያቄ ሂድ/ጂ።

3. መድሃኒቱ 50% ፍጹም ጤንነት እና 50% ወዲያው የመሞት እድል ቢኖረውስ አሁን ባሉበት ሁኔታ ለመቆየት ይፈልጋሉ ወይስ መድሃኒቱን ይወስዳሉ?

    ሀ. መድሃኒቱን ከመረጡ ወደ 4ተኛው ጥያቄ ሂድ/ጂ።

    ለ. አሁን ባሉበት ሁኔታ ለመቆየት ከመረጡ የመድሃኒቱን ውጤት ወደ 60% ፍጹም ጤንነት እና 40% ወዲያው የመሞት እድል ጨምር/ሪ

    - እርግጠኛ ካልሆኑ ወደ አማካይ ነጥብ ሂድ/ጂ፡- በ 55% ፍጹም ጤንነት እና 45% ወዲያው የመሞት እድል ጀምር/ሪ
        - መድሃኒቱን ከመረጡ ወደ 54% ፍጹም ጤንነት እና 46% ወዲያው የመሞት እድል ሂድ/ጂ።
        - አሁን ባሉበት ሁኔታ ለመቆየት ከመረጡ የመድሃኒቱ ውጤት ወደ 56% ፍጹም ጤንነት እና 44% ወዲያው የመሞት እድል ይሄዳል
        - በተመሳሳይ ሁኔታ ቀጥል/ይ

4. መድሃኒቱ 40% ፍጹም ጤንነት እና 60% ወዲያው የመሞት እድል ቢኖረውስ አሁን ባሉበት ሁኔታ ለመቆየት ይፈልጋሉ ወይስ መድሃኒቱን ይወስዳሉ?

    - እርግጠኛ ካልሆኑ ወደ አማካይ ነጥብ ሂድ/ጂ፡- በ 45% ፍጹም ጤንነት እና 55% ወዲያው የመሞት እድል ጀምር/ሪ
        - መድሃኒቱን ከመረጡ ወደ 44% ፍጹም ጤንነት እና 56% ወዲያው የመሞት እድል ሂድ/ጂ።
        - አሁን ባሉበት ሁኔታ ለመቆየት ከመረጡ የመድሃኒቱ ውጤት ወደ 46% ፍጹም ጤንነት እና 54% ወዲያው የመሞት እድል ይሄዳል
        - በተመሳሳይ ሁኔታ ቀጥል/ይ

… በተመሳሳይ ሁኔታ ቀጥል/ይ

ተሳታፊዎች ሃሳብ መቀየር ይፈቀድላቸዋል ። እርግጠኛ ከሆኑ 10% በማከል ከፍ ወዳለው ዩቲሊቲ ነጠብ ቀጥል/ይ እና እርግጠኛ ሳይሆኑ ሲቀሩ በ5% ከዚያም በ 1% ጭማሪ ወደ ሁዋላ እና ወደፊት በምሳሌው ላይ በተሰጠው መሰረት ቀጥል/ይ።

© 2022 Chung KY et al. *JAMA Network Open.*

eAppendix 3. List of Included Proxy Ethnicities

Proxy ethnicity	No.
Agaw	1
Alaba	1
Amhara	95
Argobba	1
Ashewa Meda	1
Gamo	2
Gurage	29
Hadiya	2
Holeta	1
Kambaata	3
Kefa	1
Oromo	98
Shinasha	4
Silt'e	16
Somali	1
Tigrinya	8
Welayta	4
Not Listed/ Did not Say	44
Total	312

© 2022 Chung KY et al. *JAMA Network Open.*

eAppendix 4. Supplemental Results

Variable Selection for Multivariable Regression from the Patient-Proxy Perspective Cleft Type

Children may have isolated cleft lip, which is less severe than isolated cleft palate, both of which are less severe than combined cleft lip and palate.[1,2] This perception of cleft disease severity has been reported from a patient perspective, a proxy perspective and a societal perspective.[1,2]

Patient Age

Patient age is known to have a significant effect on proxy-reported HrQoL.[3–6] To optimize clinical outcomes, surgery to repair a cleft lip usually occurs in the first few months of life and is recommended within the first 12 months of life and surgery to repair a cleft palate is recommended within the first 18 months of life or earlier if possible.[7] Thus, patient age was recategorized based on whether they are younger or older than 18 months.

Proxy Sex, Income, Ethnicity, Geographic location

These variables are well-recognized key indicators that represent health equity and explored in other low-and middle-income countries.[8–15]

Income: Mean monthly income was a continuous variable recategorized based on the national mean income.[14]

Ethnicity: Over fifteen ethnicities were recorded in the patient-proxy sample, and given that the Oromo ethnicity is the most prevalent ethnicity, this variable was recategorized based on this ethnicity.[16]

Religion

Religion can affect the time-trade off method and may lead to more non-traders (unwilling to give up any time in exchange for quality of life).[17] It is also a significant cultural predictor o health in Ethiopia.

Education

Proxy unable to read: Education compared to no education has been previously attributed to a lower quality of life in Ethiopia in HIV, TB and cancer.[18,19]

References

1. Riff KWW, Tsangaris E, Forrest C, et al. CLEFT-Q: Detecting Differences in Outcomes among 2434 Patients with Varying Cleft Types. *Plastic and Reconstructive Surgery*. 2019;144(1). doi:10.1097/PRS.0000000000005723
2. Salomon JA, Haagsma JA, Davis A, et al. Disability weights for the Global Burden of Disease 2013 study. *The Lancet Global Health*. 2015;3(11):e712-e723. doi:10.1016/S2214-109X(15)00069-8
3. Meeske K, Katz ER, Palmer SN, Burwinkle T, Varni JW. Parent proxy–reported health-related quality of life and fatigue in pediatric patients diagnosed with brain tumors and acute lymphoblastic leukemia. *Cancer*. 2004;101(9):2116-2125. doi:https://doi.org/10.1002/cncr.20609
4. Sung L, Petrou S, Ungar WJ. *Measurement of Health Utilities in Children*. Oxford University Press; 2009. Accessed February 19, 2019. http://www.oxfordscholarship.com/view/10.1093/acprof:oso/9780199547494.001.0001/acprof-9780199547494-chapter-04
5. Sung L, Young NL, Greenberg ML, et al. Health-related quality of life (HRQL) scores reported from parents and their children with chronic illness differed depending on utility elicitation method. *Journal of Clinical Epidemiology*. 2004;57(11):1161-1166. doi:10.1016/j.jclinepi.2004.05.003
6. Sung L, Greenberg ML, Young NL, et al. Validity of a modified standard gamble elicited from parents of a hospital-based cohort of children. *Journal of Clinical Epidemiology*. 2003;56(9):848-855. doi:10.1016/S0895-4356(03)00160-4
7. CDC. Facts about Cleft Lip and Cleft Palate | CDC. Centers for Disease Control and Prevention. Published December 28, 2020. Accessed April 29, 2021. https://www.cdc.gov/ncbddd/birthdefects/cleftlip.html
8. Boujaoude M-A, Mirelman AJ, Dalziel K, Carvalho N. Accounting for equity considerations in

cost-effectiveness analysis: a systematic review of rotavirus vaccine in low- and middle-income countries. *Cost Effectiveness and Resource Allocation*. 2018;16(1):18. doi:10.1186/s12962-018-0102-2

9. Cookson R, Mirelman AJ, Griffin S, et al. Using Cost-Effectiveness Analysis to Address Health Equity Concerns. *Value in Health*. 2017;20(2):206-212. doi:10.1016/j.jval.2016.11.027
10. Lahana E, Pappa E, Niakas D. The impact of ethnicity, place of residence and socioeconomic status on health-related quality of life: results from a Greek health survey. *Int J Public Health*. 2010;55(5):391-400. doi:10.1007/s00038-010-0171-2
11. Laxy M, Teuner C, Holle R, Kurz C. The association between BMI and health-related quality of life in the US population: sex, age and ethnicity matters. *International Journal of Obesity*. 2018;42(3):318-326. doi:10.1038/ijo.2017.252
12. Wang C, Li H, Li L, Xu D, Kane RL, Meng Q. Health literacy and ethnic disparities in health-related quality of life among rural women: results from a Chinese poor minority area. *Health and Quality of Life Outcomes*. 2013;11(1):153. doi:10.1186/1477-7525-11-153
13. Wang V, Seow Y, Leng Chow W. Influence of Ethnicity on Health-Related Quality of Life of Hemodialysis Patients in Singapore. *Int J Artif Organs*. 2012;35(3):217-225. doi:10.5301/ijao.5000014
14. Sibhat SG, Fenta TG, Sander B, Gebretekle GB. Health-related quality of life and its predictors among patients with breast cancer at Tikur Anbessa Specialized Hospital, Addis Ababa, Ethiopia. *Health Qual Life Outcomes*. 2019;17(1):1-10. doi:10.1186/s12955-019-1239-1
15. Araya LT, Fenta TG, Sander B, Gebremariam GT, Gebretekle GB. Health-related quality of life and associated factors among cervical cancer patients at Tikur Anbessa specialized hospital, Addis Ababa, Ethiopia. *Health and Quality of Life Outcomes*. 2020;18(1):72. doi:10.1186/s12955-020-01319-x
16. Refugees UNHC for. Refworld | Ethiopia: Information on the Oromo Ethnic Group, the Oromo Liberation Front (OLF), and the Oromo People's Democratic Organization (OPDO). Refworld. Accessed April 29, 2021. https://www.refworld.org/docid/3decdfcf4.html
17. Jakubczyk M, Golicki D, Niewada M. The impact of a belief in life after death on health-state preferences: True difference or artifact? *Qual Life Res*. 2016;25(12):2997-3008. doi:10.1007/s11136-016-1356-9
18. Hailu T, Yitayal M, Yazachew L. <p>Health-Related Quality of Life and Associated Factors Among Adult HIV Mono-Infected and TB/HIV Co-Infected Patients in Public Health Facilities in Northeast Ethiopia: A Comparative Cross-Sectional Study</p>. Patient Preference and Adherence. doi:10.2147/PPA.S269577
19. Tefera GM, Megersa WA, Gadisa DA. Health-related quality of life and its determinants among ambulatory patients with epilepsy at Ambo General Hospital, Ethiopia: Using WHOQOL-BREF. *PLOS ONE*. 2020;15(1):e0227858. doi:10.1371/journal.pone.0227858

© 2022 Chung KY et al. *JAMA Network Open*.

Section 1.3.2

Table 3. Evaluation of the Effect of Treatment on the Visual Analogue Scale, Time Trade-Off, and the Standard Gamble: A Multivariate Regression Analysis with results from a Non-parametric Bootstrap and Multiple Imputation

	Visual Analogue Scale N=230		Time Trade-Off N=230		Standard Gamble N=228	
**Variable**	Parameter Estimate (95% CI)	Pr > |t|	Parameter Estimate (95% CI)	Pr > |t|	Parameter Estimate (95% CI)	Pr > |t|
**Intercept (Cleft Lip (CL))**	**0.62 (0.52-0.72)**	**<.0001**	**0.75(0.64-0.87)**	**<.0001**	**0.63(0.49-0.77)**	**<.0001**
Non-parametric bootstrap (200 samples)	0.62(0.50-0.74)		0.75(0.65-0.87)		0.63(0.47-0.77)	
Multiple Imputation	0.63(0.54-0.73)		0.76(0.65-0.87)		0.64(0.52-0.76)	
**TREATED (Surgery +/- Speechcare)**	**0.17(0.09-0.26)**	**0.0001**	**0.15(0.05-0.25)**	**0.005**	**0.08 (-0.05-0.20)**	**0.22**
Non-parametric bootstrap (200 samples)	0.17(0.09-0.29)		0.15(0.06-0.25)		0.09(-0.05-0.20)	
Multiple Imputation	0.16(0.09-0.24)		0.13(0.04-0.23)		0.09(-0.01-0.19)	
**Cleft Type (Cleft Palate Ref: CL)**	**-0.11(-0.20--0.02)**	**0.01**	**0.05(-0.05-0.16)**	**0.32**	**0.00(-0.12-0.12)**	**0.99**
Non-parametric bootstrap (200 samples)	-0.11(-0.21--0.01)		0.05(-0.04-0.16)		0.00(-0.11-0.11)	
Multiple Imputation	-0.08(-0.16--0.001)		0.05(-0.05-0.15)		-0.03(-0.13-0.08)	
**Cleft Type (Cleft Lip and Palate Ref: CL)**	**-0.13(-0.20--0.07)**	**<.0001**	**-0.03(-0.10-0.04)**	**0.48**	**-0.02(-0.10-0.06)**	**0.64**
Non-parametric bootstrap (200 samples)	-0.13(-0.20--0.07)		-0.03(-0.10-0.04)		-0.02(-0.11-0.07)	
Multiple Imputation	-0.09(-0.15-0.04)		-0.03(-0.09-0.04)		-0.03(-0.10-0.04)	
**Income[a] (> National Average)**	**0.10(0.02-0.17)**	**0.02**	**0.11(0.02-0.20)**	**0.02**	**0.03(-0.07-0.14)**	**0.56**
Non-parametric bootstrap (200 samples)	0.10(0.03-0.19)		0.11(0.02-0.19)		0.03(-0.08-0.15)	
Multiple Imputation	0.08(0.005-0.15)		0.11(0.03-0.20)		0.02(-0.07-0.11)	
**Religion (Christian)**	**0.02(-0.05-0.08)**	**0.62**	**-0.1(-0.17--0.03)**	**0.006**	**0.01(-0.07-0.09)**	**0.84**
Non-parametric bootstrap (200 samples)	0.02(-0.05-0.08)		-0.1(-0.16--0.03)		0.01(-0.08-0.06)	
Multiple Imputation	-0.008(-0.06-0.05)		-0.1(-0.17--0.03)		0.01(-0.06-0.09)	
**Proxy Sex**	**-0.05(-0.11-0.005)**	**0.07**	**0.02(-0.05-0.09)**	**0.57**	**0.05 (-0.03-0.13)**	**0.2**
Non-parametric bootstrap (200 samples)	-0.06(-0.11-0.004)		0.02(-0.06-0.08)		0.06(-0.02-0.13)	
Multiple Imputation	-0.05(-0.10-0.004)		0.00(-0.06-0.07)		0.04(-0.02-0.11)	
**Patient Age (>= 18 months)**	**0.01(-0.07-0.10)**	**0.78**	**-0.07(-0.17-0.03)**	**0.16**	**-0.02(-0.14-0.10)**	**0.77**
Non-parametric bootstrap (200 samples)	0.01(-0.08-0.10)		-0.07(-0.16-0.01)		-0.02(-0.16-0.10)	
Multiple Imputation	0.03(-0.04-0.11)		-0.05(-0.14-0.04)		-0.04(-0.14-0.06)	
**Education (Proxy Unable to Read)**	**-0.02(-0.11-0.07)**	**0.72**	**0.02(-0.08-0.13)**	**0.67**	**-0.08(-0.20-0.05)**	**0.21**

© 2022 Chung KY et al. *JAMA Network Open.*

Non-parametric bootstrap (200 samples)	-0.01(-0.10-0.07)		0.02(-0.08-0.11)		-0.08(-0.19-0.04)	
Multiple Imputation	-0.03(-0.10-0.04)		-0.01(-0.10-0.08)		-0.04(-0.02-0.11)	
**Location (Living outside of Addis Ababa)**	**0.02(-0.04-0.08)**	**0.56**	**-0.05 (-0.13-0.02)**	**0.15**	**-0.06(-0.14-0.03)**	**0.18**
Non-parametric bootstrap (200 samples)	0.02(-0.05-0.09)		-0.05(-0.11-0.02)		-0.05(-0.14-0.03)	
Multiple Imputation	0.001(-0.05-0.06)		-0.06(-0.12-0.01)		-0.05(-0.13-0.02)	
**Oromo Ethnicity**	**0.02(-0.04-0.08)**	**0.42**	**-0.02(-0.09-0.05)**	**0.5**	**-0.01(-0.09-0.07)**	**0.78**
Non-parametric bootstrap (200 samples)	0.02(-0.03-0.08)		-0.02(-0.11-0.05)		-0.01(-0.11-0.06)	
Multiple Imputation	0.03(-0.02-0.09)		-0.03(-0.10-0.03)		-0.02(-0.09-0.05)	

a Income refers to monthly income, and average refers to the mean monthly income of the sample.

© 2022 Chung KY et al. *JAMA Network Open.*

1.3.3 Figure 1 – Box-Plot Comparison of Mean Utilities based on Treatment: Independent Samples T-Test for Patient-Proxy Participants, and Paired Sample T-Test for Societal Participants with Mean Utility Estimates.

Legend

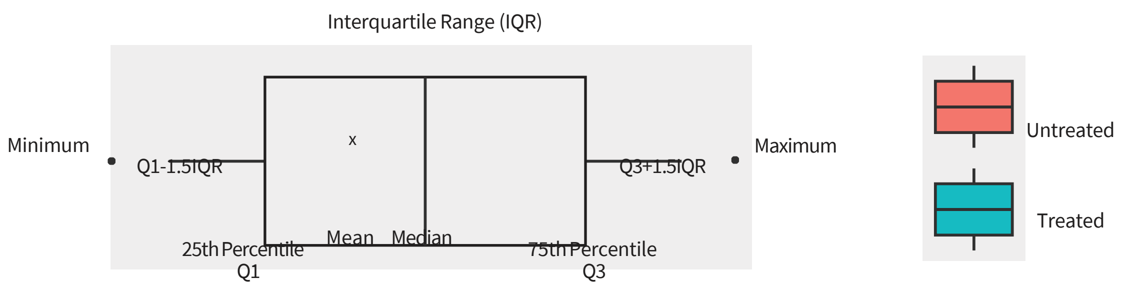

Patient-Proxy Preferences

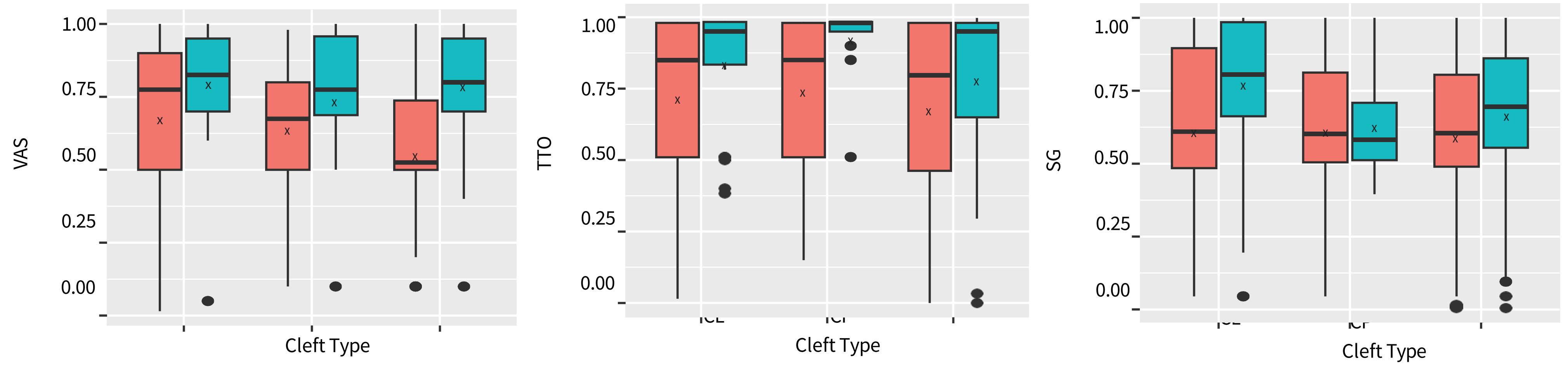

	VAS					TTO					SG				
	Untreated		Treated		p-value	Untreated		Treated		p-value	Untreated		Treated		p-value
	Mean	SD	Mean	SD		Mean	SD	Mean	SD		Mean	SD	Mean	SD	
CL	0.7	0.22	0.8	0.21	<0.05	0.73	0.29	0.84	0.21	0.086	0.62	0.28	0.77	0.26	<0.05
CP	0.64	0.21	0.75	0.24	0.13	0.75	0.26	0.93	0.12	<0.05	0.62	0.29	0.63	0.17	0.87
CLP	0.57	0.23	0.8	0.18	<.0001	0.7	0.29	0.81	0.24	<0.05	0.61	0.31	0.67	0.24	0.16

Societal Preferences

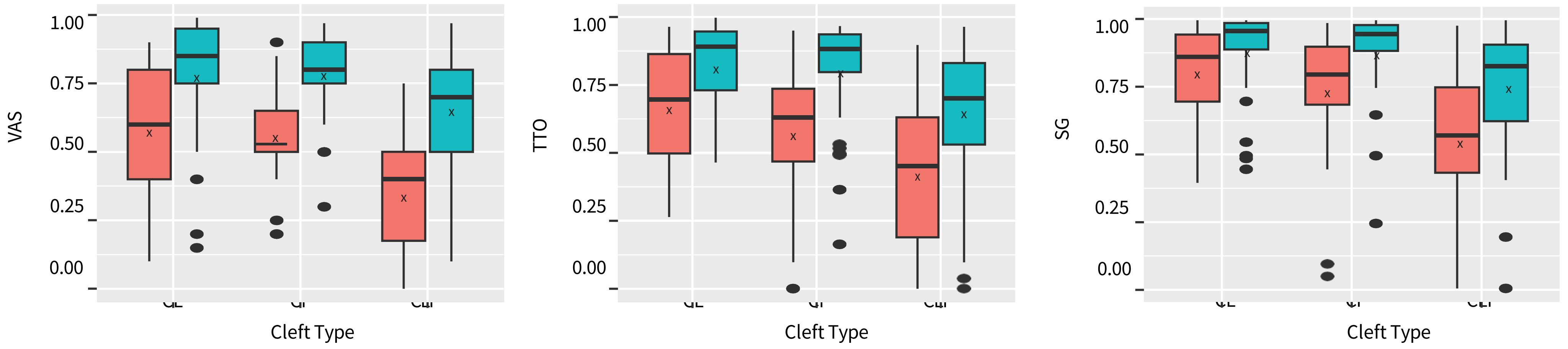

	VAS					TTO					SG				
	Untreated		Treated		p-value	Untreated		Treated		p-value	Untreated		Treated		p-value
	Mean	SD	Mean	SD		Mean	SD	Mean	SD		Mean	SD	Mean	SD	
CL	0.59	0.23	0.79	0.2	<.0001	0.68	0.21	0.83	0.15	<.0001	0.8	0.18	0.9	0.15	<.0001
CP	0.57	0.17	0.79	0.14	<.0001	0.59	0.22	0.82	0.19	<.0001	0.74	0.26	0.89	0.15	<.0001
CLP	0.35	0.21	0.64	0.2	<.0001	0.41	0.26	0.65	0.25	<.0001	0.55	0.27	0.75	0.22	<.0001

© 2022 Chung KY et al. *JAMA Network Open.*
